# ChAdOx1 nCoV-19 protection against SARS-CoV-2 in rhesus macaque and ferret challenge models

**DOI:** 10.1038/s42003-021-02443-0

**Published:** 2021-07-26

**Authors:** Teresa Lambe, Alexandra J. Spencer, Kelly M. Thomas, Karen E. Gooch, Stephen Thomas, Andrew D. White, Holly E. Humphries, Daniel Wright, Sandra Belij-Rammerstorfer, Nazia Thakur, Carina Conceicao, Robert Watson, Leonie Alden, Lauren Allen, Marilyn Aram, Kevin R. Bewley, Emily Brunt, Phillip Brown, Breeze E. Cavell, Rebecca Cobb, Susan A. Fotheringham, Ciaran Gilbride, Debbie J. Harris, Catherine M. K. Ho, Laura Hunter, Chelsea L. Kennard, Stephanie Leung, Vanessa Lucas, Didier Ngabo, Kathryn A. Ryan, Hannah Sharpe, Charlotte Sarfas, Laura Sibley, Gillian S. Slack, Marta Ulaszewska, Nadina Wand, Nathan R. Wiblin, Fergus V. Gleeson, Dalan Bailey, Sally Sharpe, Sue Charlton, Francisco J. Salguero, Miles W. Carroll, Sarah C. Gilbert

**Affiliations:** 1 The Jenner Institute, Nuffield Department of Medicine, University of Oxford, Oxford, UK; 2 National Infection Service, Public Health England, Salisbury, UK; 3 The Pirbright Institute, Woking, Surrey, UK; 4 Department of Oncology, University of Oxford, Oxford, UK; 5 Wellcome Trust Centre for Human Genetics, Nuffield Department of Medicine, University of Oxford, Oxford, UK

**Keywords:** Live attenuated vaccines, Viral infection

## Abstract

Vaccines against SARS-CoV-2 are urgently required, but early development of vaccines against SARS-CoV-1 resulted in enhanced disease after vaccination. Careful assessment of this phenomena is warranted for vaccine development against SARS CoV-2. Here we report detailed immune profiling after ChAdOx1 nCoV-19 (AZD1222) and subsequent high dose challenge in two animal models of SARS-CoV-2 mediated disease. We demonstrate in rhesus macaques the lung pathology caused by SARS-CoV-2 mediated pneumonia is reduced by prior vaccination with ChAdOx1 nCoV-19 which induced neutralising antibody responses after a single intramuscular administration. In a second animal model, ferrets, ChAdOx1 nCoV-19 reduced both virus shedding and lung pathology. Antibody titre were boosted by a second dose. Data from these challenge models on the absence of enhanced disease and the detailed immune profiling, support the continued clinical evaluation of ChAdOx1 nCoV-19.

## Abstract (web summary)

Lambe, Spencer, Thomas, Gilbert and colleagues report on the detailed immune profile of rhesus macaques and ferrets vaccinated against SARS-CoV-2 under high dose challenge. Their findings indicate that the ChAdOx1 nCoV-19 (AZD1222) the vaccine induces immune responses and reduces disease symptoms in both models, including SARS-CoV-2 mediated pneumonia and virus shedding.

## Introduction

In response to the COVID-19 pandemic, multiple candidate vaccines have entered preclinical and clinical development, and clinical efficacy has now been demonstrated for a number of vaccine platforms^1,2^. Inactivated^3,4^, adenoviral-vectored^5,6^ RNA^7^ and DNA vaccines^8,9^ have demonstrated protection against SARS-CoV-2 challenge in rhesus macaques, and SARS-CoV-2 infection has been shown to protect against rechallenge in this species^10,11^. At the start of this COVID pandemic, multiple animal SARS-CoV-2 challenges studied were initiated with the primary aim of demonstrating vaccine safety, and importantly to determine if vaccine enhanced disease was induced following vaccination and challenge. These studies were critical to enable the initiation of human clinical trials. In some early work on the development of a vaccine against SARS-CoV-1 enhanced disease including pulmonary immunopathology was induced in vaccinated animals after exposure to virus^12,13^ and it is necessary to rule out this phenomenon for novel vaccines against SARS-CoV-2. In an initial study of NHPs challenged with a high dose SARS-CoV-2, ChAdOx1 nCoV-19 was shown to be immunogenic, safe, and led to a reduction in viral load. In this present study, we have further explored the safety profile, immunogenicity, and efficacy of ChAdOx1 nCoV-19 (AZD1222) in two different animal models challenged with SARS-CoV-2. Using computerised tomography (CT) scanning we show in rhesus macaques that the changes in the lungs induced after SARS-CoV-2 challenge are similar to the changes in human lung tissue during COVID-19, and can be prevented by intramuscular vaccination with ChAdOx1 nCoV-19. In the ferret model system, vaccine-enhanced disease can be transiently induced by a formalin-inactivated alum-adjuvanted SARS-CoV-2 vaccine (FIV), resulting in increased lung pathology^14^. In the same model, here we demonstrate lung pathology was reduced following ChAdOx1 nCoV-19 vaccination and challenge when compared with animals vaccinated with ChAdOx1 vaccine expressing an irrelevant antigen (green fluorescent protein; GFP).

Importantly, this work demonstrates both safety and a reduction in disease pathology in two animal models using a new methodology to detect disease at both early and late challenge timepoints. Further evidence of a Th1 bias after vaccination with ChAdOx1 nCoV-19 is demonstrated and the immune response post vaccination with ChAdOx1 nCoV-19 and after challenge with SARS-CoV-2 is described in detail.

## Results

### Immune response to ChAdOx1 nCoV-19 vaccination in rhesus macaques and ferrets

**Fig. 1:**
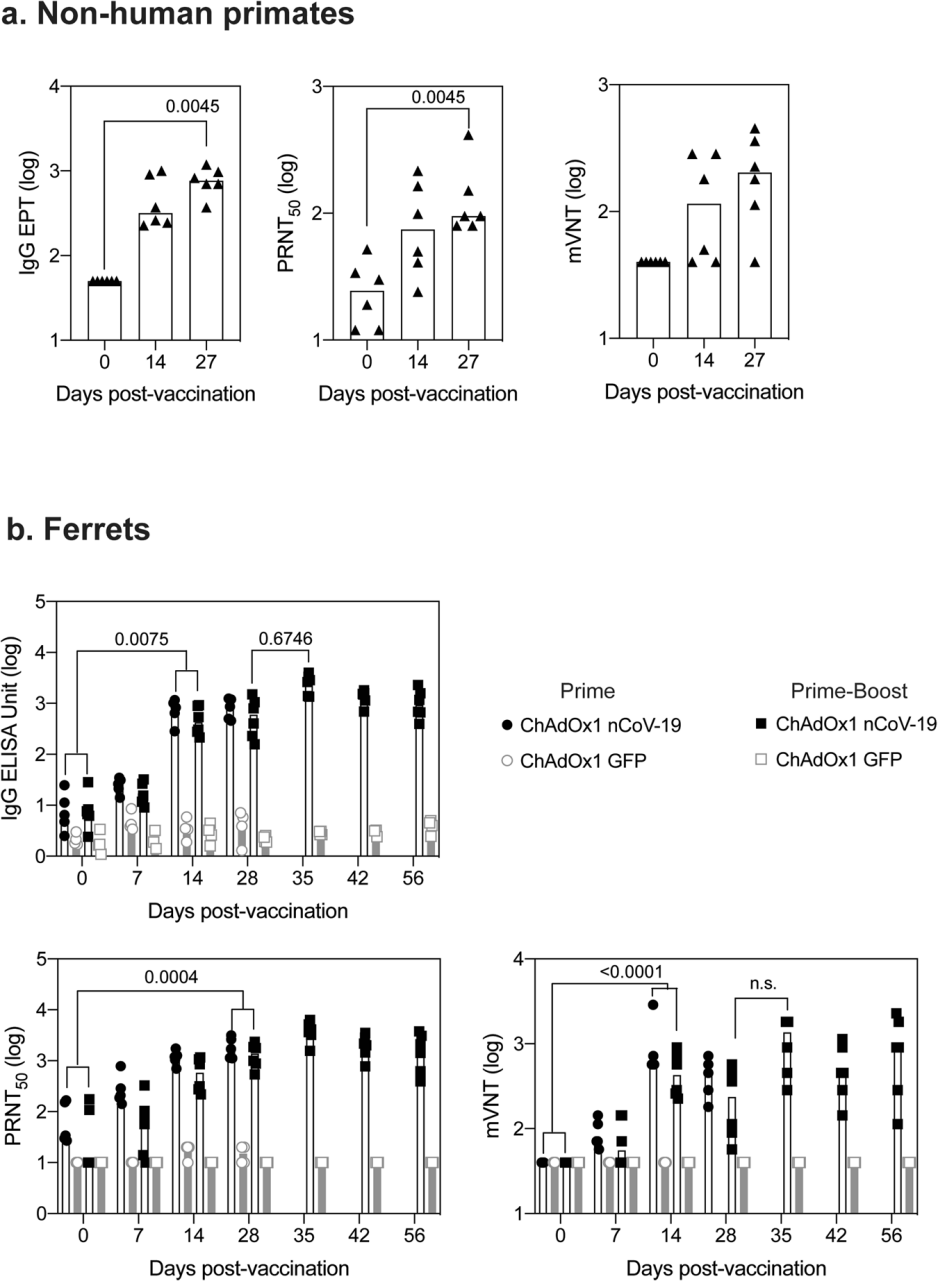
Antibody responses in rhesus macaques and ferrets following vaccination with ChAdOx1 nCoV-19. **a** Anti-spike responses, ELISA, and neutralisation titres (PRNT_50_) were measured in the serum and pseudoneutralisation titres (mVNT ID_50_) in the plasma of rhesus macaque on days 0, 14, and 27 post vaccination. Data was analysed with a Friedman one-way anova and post hoc test. Responses in rhesus macaques vaccinated with PBS were below the limit of detection. **b** Anti-spike responses, ELISA and neutralisation titres measured in the serum and pseudoneutralisation titres measured in the plasma of ferrets following vaccination with ChAdOx1 nCoV-19 or ChAdOx1 GFP. Data were analysed by a one-way anova and post hoc test comparing all ChAdOx1 nCoV-19 vaccinated to all ChAdOx1 GFP at each relevant timepoint.

ChAdOx1 nCoV-19 is a replication-deficient simian adenoviral vector expressing a codon-optimised full-length SARS-CoV-2 spike protein that has been shown to prevent SARS-CoV-2 pneumonia in rhesus macaques at a dose of 2.5 × 10^10^ vp^6^ and is immunogenic with an acceptable safety profile in humans at a dose of 5 × 10^10^ vp^15^. Here, six adult rhesus macaques (three male, three female) were vaccinated with a single dose of 2.5 × 0^10^ vp, with an equivalent control group receiving phosphate-buffered saline (PBS). Humoral immunogenicity was assessed at 14 and 27 days after vaccination by ELISA, with IgG (median titre 316 and 764 respectively) (Fig. 1a), IgM (median titre 126 and 66.7 respectively), and IgA (median titre 188 and 363 respectively) (Fig S1a) spike-specific antibodies induced in all the vaccinated animals. Neutralising antibodies were assessed in a PRNT_50_ assay, determining the antibody titre required for a 50% reduction in viral plaque formation in susceptible cells. All six vaccinated animals produced neutralising antibodies with a median titre of 74.5 (sd 76.6) at day 14 and 95 (sd 131) at day 27. (Fig. 1a). Neutralising antibodies were also assessed in a pseudoneutralization assay (Fig. 1a), with a peak in median titre of 203 (sd 154) observed at day 28 and a strong correlation (*r*^2^ = 0.4032, *p* = 0.0265) between the two neutralisation assays (Fig S1a, right).

In all ferrets immunised with a single dose of 2.5 × 10^10^ vp ChAdOx1 nCoV-19, spike-specific IgG (Fig. 1b), IgM and IgA (Fig S1b) antibodies were increased relative to animals vaccinated with control ChAdOx1 GFP vector, expressing green fluorescent protein as the vaccine antigen. Peak IgG (median 772 sd 3.36) and IgA (160 sd 3.07) titres were detected at day 28, whilst the highest post-prime IgM titre (median 378 sd 86.7) was observed at day 14. PRNT_50_ titres reached 1118 (sd 478) at day 14 and 1708 (sd 809) at day 28 (median of 11 animals) (Fig. 1b). In six ferrets receiving a second vaccine dose at day 28, PRNT_50_ titres increased from 1379 (sd 699) at day 28 to 3867 (sd 1645) at day 35 (median of six animals). These titres were not significantly higher than a single dose vaccination (Fig. 1b). Consistent with NHP antibody responses, pseudo neutralisation assays showed similar high titres post-vaccination with a median titre of 160 (sd 3.07) observed at day 28 increased to a median titre of 349 (sd 10.1) at day 35 (Fig. 1b) with strong correlation (*r*^2^ = 0.6714, *p* < 0.0001) between neutralisation assays (Fig S1b).

**Fig. 2:**
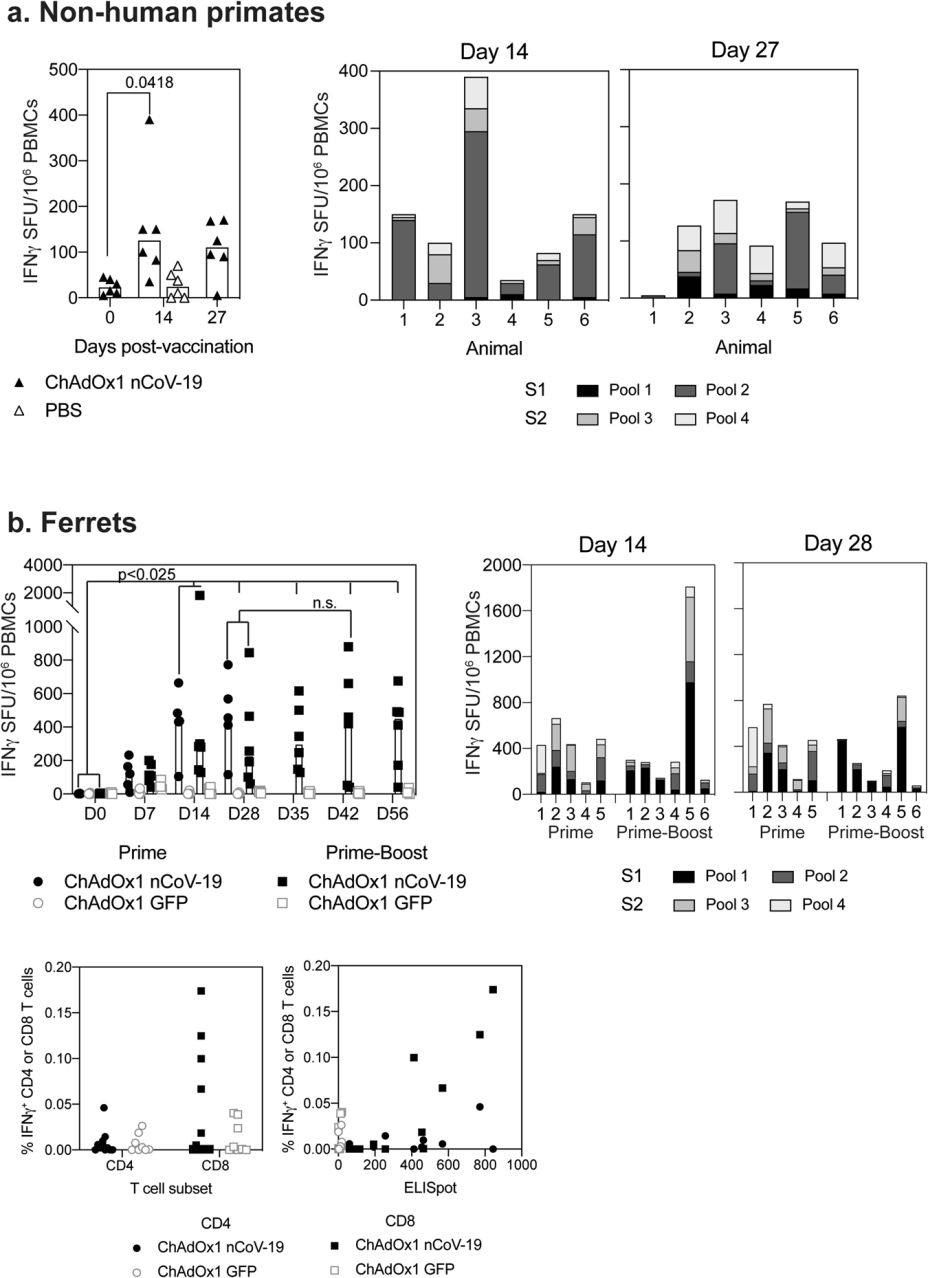
T cell responses in rhesus macaques and ferrets following vaccination with ChAdOx1 nCoV-19. Spike-specific T cell response in rhesus macaques (**a**) and ferrets (**b**) monitored by IFNγ ELISpot following vaccination and ICS (ferrets only). Response from ChAdOx1 nCoV-19 vaccinated NHPs was analysed with a Friedman one-way anova and post hoc test. Response in ferrets was analysed with a non-parametric one-way anova (Kruskal−Wallis) and post hoc Dunn’s multiple comparison test. A significant increase in the response compared to Day 0 was observed from day 14 onwards, with no statistically significant increase in the T cell response following booster vaccination. T cell responses in ferrets were measured by intracellular cytokine staining on day 28 post-vaccination and compared to responses measured by IFNγ ELISpot.

T cell responses to SARS-CoV-2 spike were also assessed by interferon-gamma ELISpot in both rhesus macaques and ferrets. A significant increase in the total spike-specific T cell response was observed at day 14 in rhesus macaques (Fig. 2a). Across the peptide pools spanning the spike protein, T cell responses were measured against all regions (Fig. 2a); however, responses were typically higher to S1 peptide pools when compared to S2, (Fig. 2a). Measurement of cytokine production in the supernatant of PMBCs stimulated with spike peptides spanning the dominant S1 region showed a log increase in IL2 levels in vaccinated animals when compared to PBS control animals (Fig S2a). In addition, an increase in IFNγ was measured. No change in IL1b, IL8, IL6, or IL10 was measured between ChAdOx1 nCoV-19 vaccinated animals and PBS control animals. Simultaneous measurement of IFNγ, IL5, or IL13 by FLUROSpot assay on day 27 rhesus macaque PBMCs, stimulated with spike peptides, demonstrated Th1 bias of the antigen-specific response with a higher number of antigen-specific IFNγ producing cells observed, compared to cells producing either IL5 or IL13. (Fig S2b).

A statistically significant (*p* = 0.001) increase in spike-specific T cells was observed in ferrets from day 14 onwards when compared to the day of vaccination (Fig. 2B), and a small but non-significant increase in IFNγ producing T cells was observed after boosting. Mapping of T cell responses across peptides spanning the spike protein, showed responses to all peptide pools, which were predominantly directed against the S1 peptide pools (pool 1 and pool 2) (Fig. 2b), with proportional responses to individual pools in each animal not changing over time (Fig S3a). At day 28 post-vaccination, antigen-specific IFNγ^+^ CD8^+^ T cell responses were detected by flow cytometry (Fig. 2b). No statistically significant difference in IFNγ^+^, TNFα^+^, or IL4^+^ CD4^+^ T cells was observed between ChAdOx1 nCoV-19 and ChAdOx1-GFP vaccinated animals on day 28 (Fig. 2b) (data not shown). Comparison of IFNγ detected by ELISpot or ICS demonstrated that CD8^+^ T cells were the predominant population producing IFNγ at day 28 post-vaccination (Fig. 2b).

### CT assessment of SARS-CoV-2 disease in vaccinated and non-vaccinated rhesus macaques after SARS-CoV-2 challenge

**Fig. 3:**
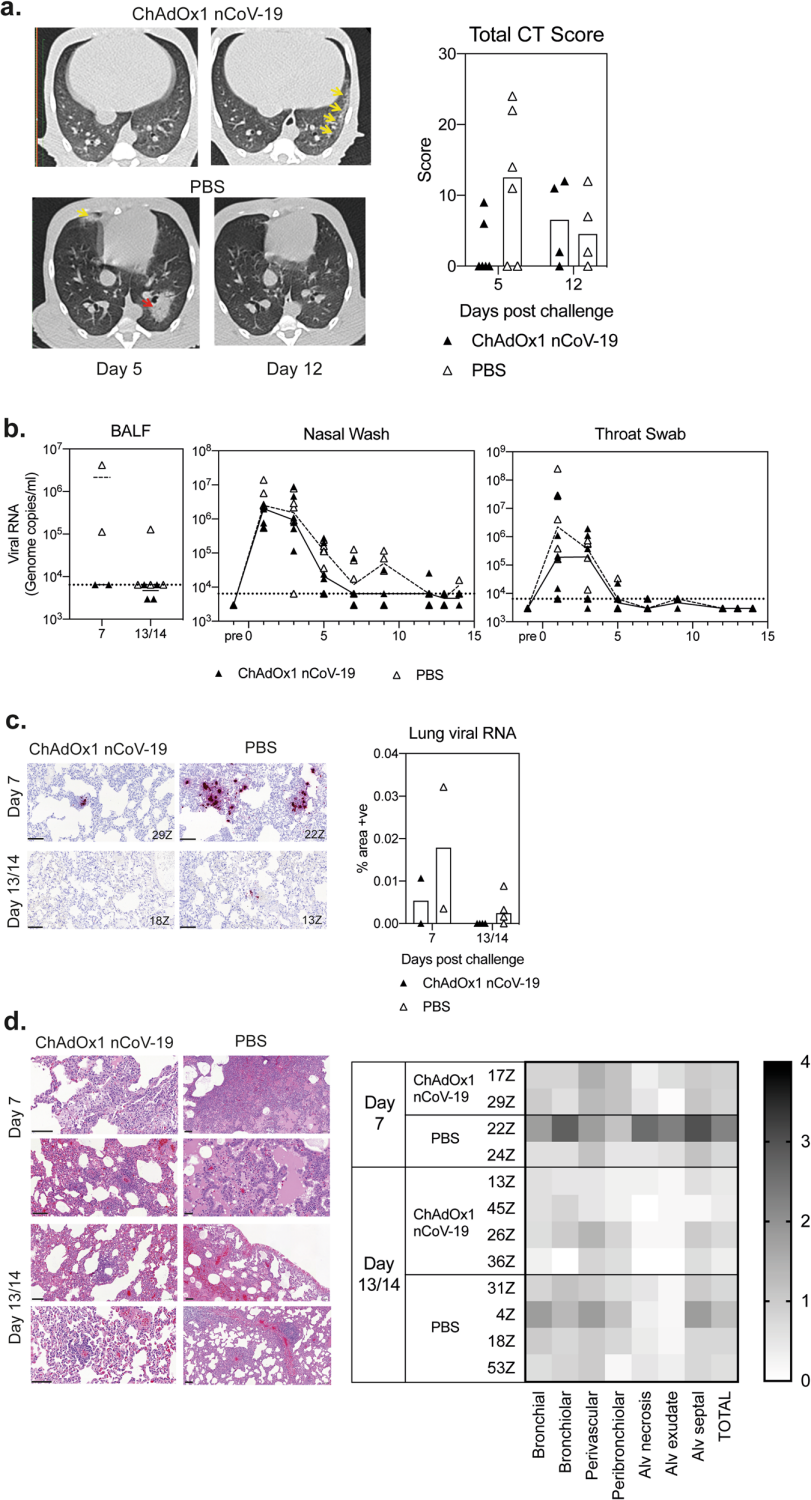
Challenge of rhesus macaques with SARS-CoV-2. **a** Representative CT scans of a vaccinated male (top panel), normal appearance at D5, unilateral mild abnormalities at D12, with peripheral ground-glass opacity (GGO) marked by yellow arrows, and PBS vaccinated female (lower panel) with bilateral disease on D5, mid-lobe GGO (yellow arrow), left lower lobe consolidated organising pneumonia pattern (red arrow), resolved by D12. The graph represents the total CT score representing disease severity. **b** Viral RNA quantitation in bronchoalveolar lavage fluid (BALF), nasal washes, and throat swabs. **c** RNA staining at day 7 (top) and day 13/14 (bottom) after challenge, the graph represents the quantification of viral RNA by ISH in the lung from all animals at 7 and 13/14 days after challenge. **d**. Histopathology at day 7 (top 2 panels), day 13/14 (bottom 2 panels) after challenge, and heatmap showing the relative frequency of histopathological abnormalities detected in different lung locations. Lines denote the size of the image, each line represents 100 μm.

Twenty-seven days after vaccination all twelve rhesus macaques were challenged with a total of 5 × 10^6^ pfu SARS-CoV-2 administered via both intratracheal and intranasal routes. CT scans were performed on all animals 15 days prior to challenge and on day 5 after challenge. Two animals per group were euthanized at day 7 for necropsy, and CT scans were performed again on day 12 in the remaining animals. Representative examples of the CT scans are shown in (Fig. 3a and S4a.). A scoring system (Table S1) was used to quantitate disease pattern and distribution (Fig. S4b and Table S2) (Supplementary Information) which was combined to produce a total score (Fig. 3a)^16^. The CT scans confirm that five days after direct instillation of SARS-CoV-2 into the trachea and nose, lung tissue became infected resulting in pathological findings similar to mild clinical cases of human COVID-19^17^. These changes were seen in four out of six PBS control and two out of six ChAdOx1 nCoV-19 vaccinated macaques, with reduced disease scores in the two vaccinated animals who demonstrated pulmonary changes (Fig. 3a).

As in human disease, higher incidence of abnormalities in the lungs was observed in males than in females. Some abnormalities were detected in all male animals on either day 5 or day 12 post challenge, but only 50% of females (Table S1). Where abnormalities were reported they were at low levels with less than 25% of the lung involved, indicating that rhesus macaques experience mild disease in this challenge model similar to mild clinical cases of human disease (Fig. S4c). There were fewer abnormal findings in vaccinated than control animals at day 5 post challenge, with equivalent amounts at day 12 (Fig. S4c). Systemic monitoring of animal health showed significantly more weight loss in control animals compared to ChAdOx1 nCoV-19 vaccinated animals (*p* = 0.0108) (Fig S4d). Over the entire post-challenge period, no statistically significant difference in body temperature between groups was observed, however at day 1 post challenge control animals had a higher median body temperature (39.4, sd 0.423) compared to vaccinees (median 38.8, sd 0.480) (Fig. S4e). Similarly, a small increase in body temperature in controls animals (median 39.050, sd 0.204) compared to ChAdOx1 nCoV-19 vaccinated animals (38.45, sd 0.383) was also observed at day 3 post challenge.

### Detection of viral RNA and histopathology following challenge of rhesus macaques

Bronchoalveolar lavage was performed at necropsy in two animals per group on days 7, 13, and 14 post-challenge. Viral RNA was only detected in bronchoalveolar lavage fluid (BALF) from control animals (Fig. 3b). Viral RNA was also quantitated in nasal wash samples and throat swabs with similar results in both groups (Fig. 3b).

Viral RNA was detected in staining of lung tissue sections in both control animals on day 7 post-challenge and three out of four control animals on day 13/14, but only one vaccinated animal, on day 7 (Fig. 3c). Lesions consistent with infection with SARS-CoV-2 were observed in the lungs of animals from both the control and vaccinated groups (Fig. 3d), with a considerably greater severity in one of the control animals. These lesions included diffuse alveolar damage, alveolar hyperplasia, perivascular and peribronchiolar lymphoid infiltrates, and bronchial/bronchiolar necrosis and exudates (Fig. 3d). No significant changes were observed in any other tissues examined. At 13/14 days post-challenge, multifocal areas of lung pathology, as described at 7 days post-challenge, together with signs of lesion resolution, were noted at reduced severity in three out of the four control animals; in the remaining animal, lesion severity had not reduced. Minimal lesions were also noted in three out of four vaccinated animals; however, in one animal, mild, multifocal interstitial pneumonia and perivascular cuffing were observed.

### Detection of virus and histopathology following challenge of ferrets

**Fig. 4:**
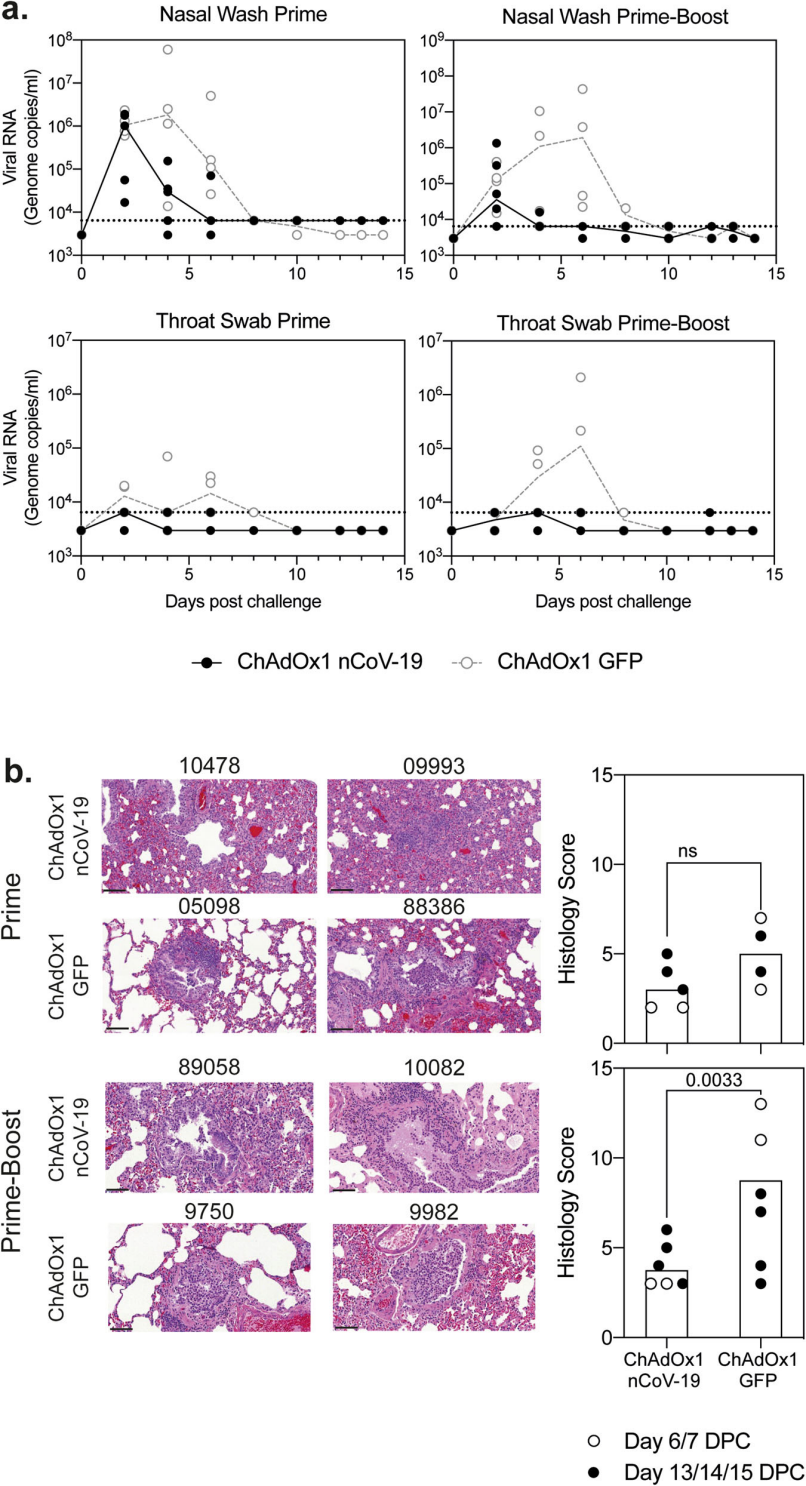
Challenge of ferrets with SARS-CoV-2. **a** Quantification of virus RNA by PCR in nasal washes and throat swabs in ferrets vaccinated with ChAdOx1 nCoV-19 (black closed) or ChAdOx1 GFP controls (grey open) following challenge with SARS-CoV-2. The limit of quantification in the assay is indicated as a dotted line on the graph. **b** Histopathology was performed on lung sections of animals culled 1 week after challenge (day 6 or 7) (presented) or 2 weeks after challenge (days 13, 14, or 15) (Fig. S5). Lines denote the size of the images, each line represents 100 μm. Graphs represent the total histopathological score of each animal. Data points represent each animal, with bars denoting the median per group. Histopathological score data in each challenge was analysed with a two-way anova to determine the effect of vaccination and day of cull as independent variables; no difference between days was observed, a significant difference between groups was observed and is denoted on the graph.

Ferrets were challenged with 5 × 10^6^ pfu SARS-CoV-2 administered intranasally 28 days after the last vaccination, and the duration of challenge was 14 days. Challenges were staggered and took place for these groups initially (ChAdOx1 nCoV-19 and ChAdOx1 GFP prime only) followed by two further groups (ChAdOx1 nCoV-19 and ChAdOx1 GFP prime-boost). Viral RNA was detected in all groups in nasal wash samples two days after challenge, with reductions in the ChAdOx1 nCoV-19 vaccinated groups by day 4 and all vaccinated animals except for one were negative at day 6 (Fig. 4a). Viral RNA in nasal washes over the total challenge period tended to be lower in the prime-boost group than prime only (Fig. 4a). In contrast, in the ChAdOx1 GFP control groups (after one or two doses of ChAdOx1 GFP) the viral RNA in the nasal washes remained above the limit of quantification (LOQ) until day 6, (Fig. 4a). Minimal viral RNA was detected in throat swabs or BALF samples in any of the groups (Fig S5a), with no virus above baseline detected in the lung of any animal (data not shown).

Histopathology was performed on two animals per group at day 6/7 post infection and the remainder at days 13/14, with scores summarised in Fig. 4B and detailed findings included in the supplementary information. Animals vaccinated with one dose of ChAdOx1 nCoV-19 did not show any remarkable change in the lungs. On day 6/7 days post-infection in the group vaccinated with only one dose of ChAdOx1 GFP, one animal had mild lesions compatible with subacute bronchopneumonia and the other had occasional minimal bronchiolar infiltrates. In ferrets receiving one dose of ChAdOx1 GFP, histopathological changes at 13/14 days post-infection were reduced compared to 6 days post-infection. Minimal changes were observed in the lungs of animals receiving two doses of ChAdOx1 nCoV-19 at either 6 or 13/14 days post-infection, and a significant increase was measured in the histology score (minimal to mild changes) in the group receiving two doses of ChAdOx1 GFP when compared to the two-dose ChAdOx1 nCoV-19 group.

### Anamnestic responses following challenge

**Fig. 5:**
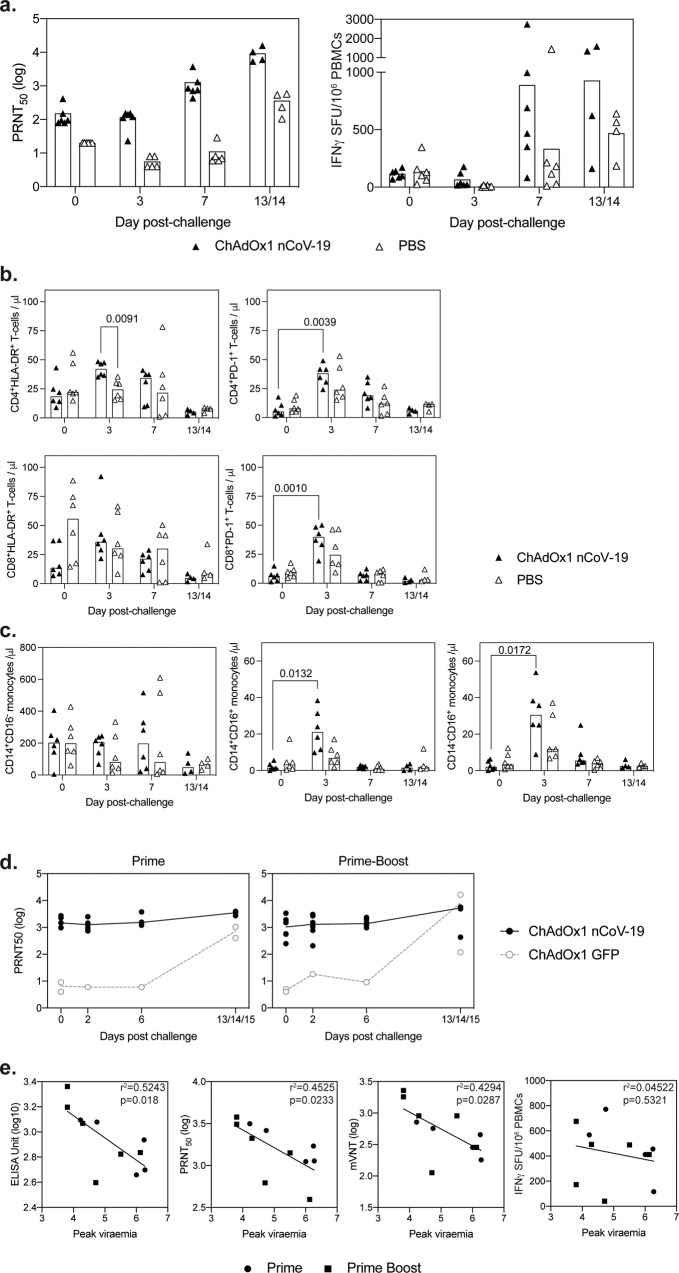
Immune responses following challenge with SARS-CoV-2. **a** Immune responses following challenge of rhesus macaques with SARS-CoV-2 were measured in virus neutralisation assays and by IFNγ ELISpot. **b** Quantification of CD4^+^ and CD8^+^ T cells expressing HLA-DR and PD-1 prior to (day 0) and at days 3, 6−7 (7) and 13−14 post SARS-CoV-2 challenge of NHPs. Data in each graph was analysed with a two-way analysis of variance (repeated measure) and a post hoc Tukey test, *p* values indicate a significant difference (*p* < 0.05) within vaccine groups over time. **c** Quantification of NHP monocyte subpopulations determined by the expression of CD14 and CD16 by whole blood immunophenotyping flow cytometry assay. Bars show group medians with values measured in individual animals shown. Data in each graph was analysed with a two-way analysis of variance (repeated measure) and a post hoc Tukey test, *p* values indicate a significant difference (*p* < 0.05) within vaccine groups over time. **d** Antibody responses in ferrets following challenge were measured in the virus neutralisation assay. **e** To determine whether antibody responses impacted on the protection of ferrets from SARS-CoV-2 infection, a Pearson correlation analysis was performed comparing peak viraemia in each ferret to IgG ELISA Unit, neutralisation titre (PRNT_50_), psuedoneutralisation titre (mVNT), or IFNγ ELISpot on the day of challenge, *r*^2^ and *p* values are indicated on each graph.

Responses were assessed in a virus IC_50_ neutralisation assay showing an increased neutralisation titre from day 3 post challenge to day 7 or day 13/14 in vaccinated non-human primates, and days 7 to 13/14 in PBS controls (Fig. 5a left). Antigen-specific cellular immune responses were measured in PBMCs stimulated with overlapping 15-mer SARS-CoV-2 spike protein-peptide pools, using an ex vivo IFN-γ ELISpot assay and also showed an increase in antigen-specific responses post-challenge in both ChAdOx1 nCoV-19 vaccinated and PBS control rhesus macaques after day 3 (Fig. 5a, right). The median response in ChAdOx1 nCoV-19 vaccinated animals was higher than that measured in the PBS control animals on day 7 and day 13/14 post-challenge (Fig. 5a, right).

Immunophenotyping flow cytometry assays were applied to whole blood samples collected immediately prior to (27 days after ChAdOx1 nCoV-19 vaccination) and at days three, seven, and 13−14 after SARS-CoV-2 challenge to explore changes in the composition and activation status of the cellular immune compartment (Fig. 5b). T cell activation status was assessed by expression of class II major histocompatibility antigen, HLA-DR, and the immune checkpoint signalling receptor PD-1. Transient increases in the number of CD4^+^ and CD8^+^ T cells expressing PD-1 were observed following SARS-CoV-2 infection in both ChAdOx1 nCoV-19 and PBS vaccinated animals (Fig. 5b). However, the number of activated HLA-DR expressing CD4^+^ and CD8^+^ T cells was significantly higher in ChAdOx1 nCoV-19 animals three days after SARS-CoV-2 challenge (*p* = 0.0039 and *p* = 0.0010 respectively), indicating that ChAdOx1 nCoV-19 vaccination had advanced the kinetics of the adaptive T cell-mediated response to infection. Similarly, the quantification of classical (CD14^+^CD16^−^), intermediate (CD14^+^CD16^+^), and non-classical (CD14^−^CD16^+^) monocyte populations revealed significant increases in immunomodulatory populations (*p* = 0.0172 intermediate; *p* = 0.0132 non-classical) in vaccinated animals suggesting that heightened early pro-inflammatory responses had been facilitated by the vaccination regimen (Fig. 5c).

In ferrets, neutralising antibodies did not dramatically increase in ChAdOx1 nCoV-19 vaccinated animals following SARS CoV-2 challenge, with both groups having similar titres of neutralising antibodies by the end of the study (Fig. 5d). In ChAdOx1 nCoV-19 vaccinated animals, the IgG antibody titres on the day of challenge, measured by ELISA, neutralisation or pseudo neutralisation assay, inversely correlated with the peak level of viraemia measured in each animal (Fig. 5e). There was no relationship between IFNγ ELISpot and peak viraemia. Overall the data would suggest that vaccine-induced protection in ferrets was not associated with the level of spike-specific T cells measured in these assays, but was associated with the humoral response to SARS-CoV-2 spike protein.

## Discussion

A safe and effective vaccine is expected to be an essential requirement to effectively control the COVID-19 pandemic. Early development of a vaccine against Feline Infectious Peritonitis, which is also caused by a coronavirus, resulted in enhanced disease in vaccinated and then challenged animals^13^, a phenomenon also seen in the early development of vaccines against SARS CoV-1^12,18^. Vaccine enhanced disease results in an increase in disease severity when vaccinated subjects are subsequently exposed to live virus. Immunopathology in coronavirus vaccinated and challenged animals has been associated with increased levels of the Th2 cytokines IL5 and IL13 and altered ratios of IgG antibody subclasses (*8,9,11-13,15*). This is similar to the vaccine-enhanced disease observed with early vaccine development against the respiratory syncytial virus (RSV); pathology was associated with a relatively high titre of non-neutralising antibodies, a role for neutrophils, eosinophils, and a predominantly a Th2-biased response was described^19–23^.

Preclinical studies of vaccines against SARS CoV-2 must therefore determine whether enhanced disease occurs in vaccinated animals once exposed to SARS-CoV-2 virus. Multiple studies of SARS-CoV-2 vaccines^3–8^ have now been conducted in rhesus macaques without demonstrating enhanced disease^24^. Using CT scanning, we show that the lung pathology associated with infection with 5 × 10^6^ pfu of SARS-CoV-2 in rhesus macaques mirrors that seen in humans with mild pneumonia caused by COVID-19, and is reduced in animals vaccinated with a single dose of ChAdOx1 nCoV-19 during the first week post-infection. Through the use of CT as an additional methodology, and sampling at later time-points post challenge we expand on the work already performed in the field and offer novel insight using a technology widely used to describe the human disease. Additionally, we expand on previous work describing disease progression and vaccine-induced protection in two animal models. Histopathology performed on the lung tissues also indicated that the lesions observed in vaccinated animals are less severe than in controls at 7 days post-challenge, consistent with our earlier study^6^, but also at a later timepoint 13/14 days post-challenge. The presence of viral RNA (using a probe that does not allow determination of viral replication) by ISH is also less frequent in vaccinated animals. Importantly the inclusion of a later timepoint after challenge and the use of CT imaging supports previously published preclinical work to definitively answer questions related to vaccine-enhanced disease in this setting.

We demonstrate in the ferret model that virus shedding early after challenge with 5 × 10^6^ pfu of SARS-CoV-2 was reduced in ChAdOx1 nCoV-19 vaccinated animals. A second vaccination with ChAdOx1 nCoV-19 increased antibody titres in ferrets. The level of spike antibodies induced by either prime or prime-boost vaccination regimens were shown to negatively correlate with the total virus shed in nasal washes. A range of antibody titres against the spike protein has been demonstrated in individuals who have had severe disease requiring hospitalisation, mild to moderate disease, and asymptomatic infection. It is unclear what level of antibody titres against the viral spike protein is required to prevent infection or avert disease but it is generally accepted that high-titre neutralising antibodies are required.

Both animal models in this study confirmed the safety of vaccination with ChAdOx1 nCoV-19 when the respiratory tract is exposed to very large quantities (5 × 10^6^ pfu) of SARS-CoV-2 virus compared to other challenge studies^24^. Reduced lung pathology, as well as reduced nasal virus shedding in ferrets, was also shown to correlate negatively with the neutralising antibody titre induced by vaccination. Here, rhesus macaques were challenged by simultaneous virus instillation to both the upper and lower respiratory tract, as in some, but not all other vaccination and challenge studies^5–8^. Ferrets were challenged by the intranasal route only, but with the animal held vertically allowing some of the inoculum to enter the lungs. Both methods, therefore, result in immediate exposure of the lungs to SARS-CoV-2, whereas unless exposed to an extremely high concentration of virus the majority of human infections are likely to infect the upper respiratory tract initially, moving to the lungs if the infection is not rapidly controlled. Differences in virus quantification in the URT and LRT in the different animal models underscore the importance of using more than one model before progression to clinical trials. The lack of disease and LRT infection in Rhesus Macaques supports the outcomes measured in clinical trials and real-world effectiveness data demonstrating that ChAdOx1 nCoV-19 is highly effective against severe disease, but occasionally mild and asymptomatic infection can occur post-vaccination^25,26^. These data and in particular, the data from the rhesus macaque animal model underscore the importance of suitable animal models to support vaccine development and clinical trial assessment.

Currently, there are no defined correlates of protection against COVID-19 infection in humans, and the immunological thresholds required for vaccine efficacy remain undefined^10^. In a rhesus macaque SARS-CoV-2 infection model, protection against re-challenge was associated with immunologically-mediated control of infection with both neutralising and non-neutralising antibody as well as cellular responses increasing after secondary viral exposure^15^. In addition, efficacy assessment of Ad26 COVID vaccine in a preclinical setting showed higher virus neutralisation titres were associated with reduced viral load following single^5^ or two-dose vaccination regimens^27^. In this current study where animals received ¼ of the vaccine and 100 times higher challenge dose, a correlation between antibody levels and reduced levels of the virus was observed in ferrets. An increasing number of reports from human studies are demonstrating strong and early T cell and B cell response are associated with better disease outcome^28^. Taking all this data together it is therefore suggested that high titre neutralising antibodies with a robust cytotoxic CD8^+^ T cell response and Th1 biased CD4^+^ effector response will be optimal for protective immunity following SARS-CoV-2 exposure, as demonstrated here. Viral vectored vaccines have been demonstrated to induce strong immune responses in older adults and immunocompromised individuals and have been used in repeat vaccinations, subsequently inducing strong cellular and humoral immunity^29–32^. ChAdOx1 nCoV-19 vaccination has previously been demonstrated to prevent early SARS-CoV-2 mediated pneumonia in rhesus macaques^6^, and this work is further supported and extended by the studies presented here.

## Methods

### Animals

Twelve rhesus macaques of Indian origin (Macaca mulatta) were used in this study. Study groups comprised three males and three females and all were adults aged 4 years and weighing between 4.30 and 8.24 kg at the time of challenge. Before the start of the experiment, socially compatible animals were randomly assigned to challenge groups, to minimise bias.

Animals were housed in compatible social groups, in cages in accordance with the UK Home Office Code of Practice for the Housing and Care of Animals Bred, Supplied or Used for Scientific Procedures (2014) and National Committee for Refinement, Reduction and Replacement (NC3Rs) Guidelines on Primate Accommodation, Care and Use, August 2006. Prior to challenge, the animals were housed at Advisory Committee on Dangerous Pathogens (ACDP) level two in cages approximately 2.5 M high by 4 M long by 2 M deep, constructed with high-level observation balconies and with a floor of deep litter to allow foraging. Following challenge, animals were transferred to ACDP Level three and housed in banks of cages of similar construction placed in directional airflow containment systems that allowed group housing and environmental control whilst providing a continuous, standardised inward flow of fully conditioned fresh air identical for all groups. Additional environmental enrichment was afforded by the provision of toys, swings, feeding puzzles, and DVDs for visual stimulation. In addition to ad libitum access to water and standard old-world primate pellets, the diet was supplemented with a selection of fresh vegetables and fruit. All experimental work was conducted under the authority of a UK Home Office approved project license that had been subject to local ethical review at PHE Porton Down by the Animal Welfare and Ethical Review Body (AWERB) and approved as required by the Home Office Animals (Scientific Procedures) Act 1986. Animals were sedated by intramuscular (IM) injection with ketamine hydrochloride (Ketaset, 100 mg/ml, Fort Dodge Animal Health Ltd, Southampton, UK; 10 mg/kg) for procedures requiring removal from their housing. None of the animals had been used previously for experimental procedures. Twenty-eight healthy, female ferrets (Mustela putorius furo) aged 5–7 months were obtained from a UK Home Office accredited supplier (Highgate Farm, UK). The mean weight at the time of challenge was 973 g/ferret (range 825−1129 g). Ferrets were housed in pairs at Advisory Committee on Dangerous Pathogens (ACDP) containment level 3. Cages met with the UK Home Office ‘Code of Practice for the Housing and Care of Animals Bred, Supplied or Used for Scientific Procedures’ (December 2014). Access to food and water was ad libitum and environmental enrichment was provided^33^. All experimental work was conducted under the authority of a UK Home Office-approved project licence that had been subject to local ethical review at PHE Porton Down by the Animal Welfare and Ethical Review Body (AWERB). One animal in the ChAdOx1 nCoV-19 prime only group steadily lost weight from arrival at the facility (5 days prior to vaccination) and throughout the post-vaccination follow-up and was sacrificed on welfare grounds on day 14 of the study. As the weight loss was observed from arrival it was not deemed vaccine-related, therefore all immunological data from this animal has been excluded from the analysis.

### Vaccinations

Rhesus macaques received 2.5 × 10^10^ vp ChAdOx1 nCoV-19 administered in 100μl intramuscularly or received 100μl of phosphate-buffered saline (PBS) intramuscularly and were challenged with SARS-CoV-2 twenty-seven days later. Ferrets were randomly assigned to ChAdOx1 nCoV-19 and ChAdOx1 GFP vaccinated groups. An identifier chip (Bio-Thermo Identichip, Animalcare Ltd, UK) was inserted subcutaneously into the dorsal cervical region of each animal. Ferrets were immunised with 2.5 × 10^10^ virus particles of ChAdOx1 nCoV-19 or ChAdOx1 GFP intramuscularly administered as a 100 ml volume into the hind leg. Twenty-eight days after vaccination, half of the vaccinated animals were challenged with SARS-CoV-2, while the other half received a booster dose of ChAdOx1 nCoV-19 of ChAdOx1 GFP and were challenged with SARS-CoV-2 a further twenty-eight days later.

### Enzyme-linked immunosorbent assay

Maxisorp plates (Nunc) were coated overnight at 4 °C with 250 ng/well spike protein in PBS, prior to blocking with 100 µl of casein in PBS (Thermo Fisher) for 1 h at RT. NHP serum was serially diluted 2× in casein in PBS was incubated at RT for 1 h. Antibodies were detected using affinity-purified polyclonal antibody alkaline phosphatase-labelled goat-anti-monkey IgG (Rocklands Laboratories) (1 in 10 000), anti-monkey IgM (Rockland Laboratories) (1 in 5000), or anti-monkey IgA (Rockland Laboratories) (1 in 2000) in casein and developed with NPP-substrate (Sigma) and read at 405 nm. All wells were washed at least 3× with PBST 0.05% tween in-between steps. Endpoint titre was calculated as follows: the log_10_ OD against log_10_ sample dilution was plotted and a regression analysis of the linear part of this curve allowed calculation of the endpoint titer with an OD of three times the background. Ferret serum was diluted in casein and incubated at RT for 2 h. Antibodies were detected using affinity-purified polyclonal antibody HRP-labelled goat-anti-ferret IgG (Abcam) (1 in 10 000) in casein and TMB highest sensitivity (Abcam), developed for 12 m, and the reaction was stopped using H_2_SO_4_ and read at 450 nm. Anti-spike IgM or IgA antibodies were detected with alkaline phosphatase-conjugated anti-ferret IgM (Rockland Laboratories) (1 in 2500) or anti-ferret IgA (1 in 500) (Sigma), developed with NPP-substrate and read at 405 nm. All wells were washed at least 3× with PBST 0.05% tween in-between steps. Ferret samples were run against a standard positive pool of serum generated from ChAdOx1 nCoV-19 vaccinated ferrets with high endpoint titre. Due to high levels of non-specific responses, the background was defined as the mean + 2× stdev of all animals at day 0.

### Plaque reduction neutralisation assay

Heat-inactivated (56 °C for 30 min) serum samples were serially diluted and incubated with approximately 60 PFU of wild-type SARS-CoV-2 (2019-nCoV/Victoria/1/2020), for 1 h at 37 °C in 5% CO_2_. Samples were then incubated with Vero E6 [Vero 76, clone E6 (ECACC 85020206), European Collection of Authenticated Cell Cultures, UK] monolayers in 24-well plates (Nunc, ThermoFisher Scientific, Loughborough, UK) under MEM (Life Technologies, California, USA) containing 1.5% carboxymethylcellulose (Sigma), 5% (v/v) foetal calf serum (Life Technologies) and 25 mM HEPES buffer (Sigma). After incubation, at 37 °C for 96 h, plates were fixed overnight with 20% (w/v) formalin/PBS, washed with tap water, and stained with methyl crystal violet solution (0.2% v/v) (Sigma). The neutralising antibody titres were defined as the serum dilutions resulting in a 50% reduction relative to the total number of plaques counted without antibody by using Probit analysis written in R programming language for statistical computing and graphics. An internal positive control for the PRNT assay was run using a sample of human MERS convalescent serum known to neutralise SARS-CoV-2 (National Institute for Biological Standards and Control, UK).

### Micro neutralisation test (mVNT) using lentiviral-based pseudotypes bearing the SARS-CoV-2 Spike

Lentiviral-based SARS-CoV-2 pseudotyped viruses were generated in HEK293T cells incubated at 37 °C, 5% CO_2_ as previously described^34^. Briefly, cells were seeded at a density of 7.5 *×* 10^5^ in six-well dishes, before being transfected with plasmids as follows: 500 ng of SARS-CoV-2 spike, 600 ng p8.91 (encoding for HIV-1 gag-pol), 600 ng CSFLW (lentivirus backbone expressing a firefly luciferase reporter gene), in Opti-MEM (Gibco) along with 10 µL PEI (1 µg/mL) transfection reagent. A ‘no glycoprotein’ control was also set up using the pcDNA3.1 vector instead of the SARS-CoV-2 S expressing plasmid. The following day, the transfection mix was replaced with 3 mL DMEM with 10% FBS (DMEM-10%) and incubated for 48 and 72 h, after which supernatants containing pseudotyped SARS-CoV-2 (SARS-CoV-2 pps) were harvested, pooled, and centrifuged at 1,300 x *g* for 10 m at 4 °C to remove cellular debris. Target HEK293T cells, previously transfected with 500 ng of a human ACE2 expression plasmid (Addgene, Cambridge, MA, USA) were seeded at a density of 2 × 10^4^ in 100 µL DMEM-10% in a white flat-bottomed 96-well plate one day prior to harvesting SARS-CoV-2 pps. The following day, SARS-CoV-2 pps were titrated 10-fold on target cells, and the remainder stored at −80 °C. For mVNTs, NHP plasma was diluted 1:10 and ferret plasma diluted 1:20 in serum-free media, and 50 µL was added to a 96-well plate in triplicate and titrated two-fold. A fixed titred volume of SARS-CoV-2 pps was added at a dilution equivalent to 10^5^ signal luciferase units in 50 µL DMEM-10% and incubated with sera for 1 h at 37 °C, 5% CO_2_ (giving a final sera dilution of 1:40). Target cells expressing human ACE2 were then added at a density of 2 *×* 10^4^ in 100 µL and incubated at 37 °C, 5% CO_2_ for 72 h. Firefly luciferase activity was then measured with BrightGlo luciferase reagent and a Glomax-Multi^+^ Detection System (Promega, Southampton, UK). Pseudotyped virus neutralisation titres were expressed as a 50% neutralisation dose (ND_50_) using a Spearman and Karber formula.

### ELISpot

PBMCs from rhesus macaques and ferrets were isolated from whole blood by layering over Lymphoprep (density 1.077 g) and centrifugation for 30 m at 1000 g. PBMCs were collected from the interface, washed with Hanks Balanced Salt Solution (HBSS) prior to resuspension in complete media (RPMI supplemented with 10% FCS, Pent-Strep, L-Glut and Hepes). IFNγ ELISpot assay was performed using NHP IFNγ (Mabtech) or Ferret IFNγ ELISpot^BASIC^ Kit according to the manufacturer’s protocol (MABtech). PBMCs were plated at a concentration of 250 000 cells per well (NHPs) or 100 000 cells per well (Ferrets) and were stimulated overnight (18−20 h) with four contiguous peptide pools spanning the length of the SARS-CoV-2 spike protein sequence at a concentration of 2 µg/mL per peptide (Mimotopes) (Table S7). Spots were counted and analysed on an AID ELISpot Reader (AID). Spot forming units (SFU) per 1.0 × 10^6^ PBMCs were summed across the four peptide pools for each animal after subtraction of background response (media and PBMC only wells). Simultaneous production of IFNγ, IL13, and IL5 was detected with a custom FLUROspot^FLEX^ kit (Mabtech) using anti-monkey IFNγ FluroSpot set 490, anti-monkey IL13 FluroSpot set 550 and anti-human IL5 FluroSpot set 640. ELISpot was performed with the same stimulation conditions as above (200 000 cells and four peptide pools), with plates developed according to the manufacturer’s instructions. The spot was enumerated using Mabtech IRIS^TM^ reader and analysed with SpotReader software (Mabtech). Post-challenge NHP ELISpot was performed on PBMCs isolated over a Ficoll-Paque Plus (GE Healthcare, USA) density gradient and anti-human/simian IFNγ kit (Mabtech). 200 000 cells per well were stimulated with three pools of SARS-CoV-2 peptides (Table S7, Pool 1 peptides 1−96, Pool 2 peptides 97−192, Pool 3 peptides 193−316) at a final concentration of 1.7 µg/ml, Phorbol 12-myristate (Sigma-Aldrich Dorset, UK) (100 ng/ml) and ionomycin (CN Biosciences, Nottingham, UK) (1 mg/ml) were used as a positive control. ELISpot plates were analysed using the CTL scanner and software (CTL, Germany) and further analysis was carried out using GraphPad Prism (version 8.0.1) (GraphPad Software, USA).

### Measurement of NHP serum cytokines

Rhesus macaque PBMCs were stimulated for 16 h with two pools of SARS-CoV-2 peptides (S1 and S2) and cytokine measured using MescoScaleDiscovery (MSD) Technology V-PLEX Proinflammatory Panel 1 NHP kit according to the manufacturer’s instructions. Log_10_ Fold Change (Log_10_FC) was calculated by dividing concentration detected in stimulated wells by unstimulated wells, baseline detectable level of each cytokine was set at 0.01 pg.

### Intracellular cytokine staining

Ferret PBMCs were stimulated for 18−20 h with two pools of SARS-CoV-2 spike peptides (S1-pool 1 and pool 2 or S2-pool 3 and pool 4) at a final concentration of 2 µg/ml or ConA in the presence of golgi-stop (BD) and golgi-plug (BD). Cells were surface stained with anti-mouse/rat/human CD3 Alexa 405 (clone PC3/188A, 1 in 10 dilutions) (Santa Cruz Biotechnology), anti-human CD8 APCCy7 (clone OKT8, 1 in 10 dilutions) (Thermofisher), and live-dead aqua (Thermofisher), fixed with Fix-Perm solution prior to intracellular staining with anti-bovine IFNγ PE (clone CC302, 1 in 10 dilutions) (Abserotec) and anti-mouse TNFa A647 (clone MP6-XT22, 1 in 25 dilutions). Data were acquired on a BD Fortessa and analysed in FlowJo version 9 or above. Antigen-specific T cells were identified by gating on LIVE/DEAD negative, doublet negative (FSC-H vs FSC-A), size (FSC-A vs SSC), CD3^+^, then CD4^+^ or CD8^+^ cells and IFNγ^+^ (Fig. S6). Data is presented total spike response, by summing together the frequency of cytokine positive cells detected in S1 and S2 stimulated wells after background subtraction of media stimulated cells.

### Challenge

Animals were challenged with SARS-CoV-2 (VERO/hSLAM cell passage 3 (Victoria/1/2020)) at a final challenge dose of 5 × 10^6^ pfu. NHPs received 2 ml intratracheally followed by 1 ml intranasally, ferrets received a total of 1 ml split equally between both nares.

Prior to challenge ferrets were sedated by intramuscular injection of ketamine/xylazine (17.9 and 3.6 mg/kg bodyweight). Challenge virus prepared in line with previous studies^16,33^ was delivered by intranasal instillation (1.0 mL total, 0.5 mL per nostril) diluted in phosphate-buffered saline (PBS). Nasal washes were obtained by flushing ferret nasal cavities with 2 mL PBS. Throat swabs were collected using a standard swab (Sigma Virocult®) gently stroked across the back of the pharynx in the tonsillar area. Throat swabs were processed, and aliquots were stored in viral transport media (VTM) and AVL at ≤ −60 °C until assay. Clinical signs of disease were monitored and necropsy procedures were performed in line with the previous studies^33^.

### Computed tomography (CT) radiology of NHPs

CT scans were collected from sedated macaques using a 16 slice Lightspeed CT scanner (General Electric Healthcare, Milwaukee, WI, USA) in the prone and supine position. The change in position assists differentiation between pulmonary changes due to gravity dependant atelectasis from ground-glass opacity at the lung bases caused by COVID. All axial scans were performed at 120 KVp, with Auto mA (ranging between 10 and 120), and were acquired using a small scan field of view. The rotation speed was 0.8 s. Images were displayed as an 11 cm field of view. To facilitate full examination of the cardiac/pulmonary vasculature, lymph nodes, and extrapulmonary tissues, Niopam 300 (Bracco, Milan, Italy), a non-ionic, iodinated contrast medium, was administered intravenously (IV) at 2 ml/kg body weight and scans collected immediately after injection and 90 s from the mid-point of injection. Scans were evaluated by an expert thoracic radiologist, blinded to the animal’s treatment and clinical status for the presence of COVID disease features: ground-glass opacity (GGO), consolidation, crazy paving, nodules, peri-lobular consolidation; distribution—upper, middle, lower, central 2/3, peripheral, bronchocentric) and for pulmonary embolus.

The extent of lung involvement was estimated (<25%, 25−50%, 51−75%, 76−100%) and quantified using a scoring system developed for COVID disease, as follows:

*COVID disease pattern*: Nodule(s): Score 1 for 1, 2 for 2 or 3, 3 for 4 or more. GGO: Score 1 if measures < 1 cm, 2 if 1 to 2 cm, 3 if 2−3 cm, 4 if >3 cm. Consolidation Score: 2 if measures < 1 cm, 4 if 1−2 cm, 6 if 2−3 cm, 8 if >3 cm. *Zone classification:* Each side of the lung was divided (from top to bottom) into three zones: The upper zone (above the carina), the middle zone (from the carina to the inferior pulmonary vein), and the lower zone (below the inferior pulmonary vein). Each zone was then divided into two areas: the anterior area (the area before the vertical line of the midpoint of the diaphragm in the sagittal position) and the posterior area (the area after the vertical line of the mid-point of the diaphragm in the sagittal position). This results in 12 zones in total. *Measures:* COVID pattern score = Nodule score + GGO score + consolidation score. Distribution (Zone) score = number of zones with disease, maximum score 12. Total CT score = COVID pattern score + Distribution (zone) score.

### Whole blood immunophenotyping

Assays were performed using 50 µl of heparinised blood incubated for 30 m at room temperature with optimal dilutions of the following antibodies: anti-CD3-AF700 (clone SP34-2, 1.25 μl per sample), anti-CD4-APC-H7 (clone L200, 10 μl per sample), anti-CD8-PerCP-Cy5.5 (clone SK1, 5 μl per sample), anti-HLA-DR-BUV395 (clone G45-5, dilution 2.5 μl), anti-CD25-FITC (clone M-A251, 20 μl per sample) (all from BD Biosciences, Oxford, UK); anti-CD14-PE (clone M5E2, dilution 5 μl) (Beckman Coulter); anti-CD16-BV785 (clone 3G8, dilution 5 μl), anti-CD20-PE-Dazzle (clone 2H7, 2.5 μl per sample), anti-CD95-PE-Cy7 (clone DX5, 5 μl per sample), anti-CD279(PD1)-BV711 (clone EH12-2H7, 5 μl per sample), anti-γδ-TCR-BV421 (clone TCR, 5 μl per sample) (all from BioLegend); and amine-reactive fixable viability stain red (Life Technologies); all prepared in brilliant stain buffer (BD Biosciences). Red blood cell contamination was removed using a Utilyse reagent kit as per the manufacturer’s instructions (Agilent). BD Compbeads (BD Biosciences) were labelled with the above fluorochromes for use as compensation controls. Following antibody labelling, cells and beads were fixed in a final concentration of 4% paraformaldehyde solution (Sigma Aldrich, Gillingham, UK) prior to flow cytometric acquisition. Cells were analysed using a five laser LSRII Fortessa instrument (BD Biosciences) and data were analysed using FlowJo (version 9.7.6, BD Biosciences). Immediately prior to flow cytometric acquisition, 50 µl of Truecount bead solution (Beckman Coulter) was added to each sample. Leucocyte populations were identified using a forward scatter-height (FSC-H) versus side scatter-area (SSC-A) dot plot to identify the lymphocyte, monocyte, and granulocyte populations, to which appropriate gating strategies were applied to exclude doublet events and non-viable cells (Fig S7). Lymphocyte subpopulations including T-cells, NK-cells, NKT-cells, and B-cells were delineated by the expression pattern of CD3, CD20, CD95, CD4, CD8, CD127, CD25, CD16, and the activation and inhibitory markers HLA-DR and CD279 (PD-1). Classical- and non-classical-monocytes were identified by the expression pattern of HLA-DR, CD14, and CD16. Granulocyte populations were delineated into neutrophils and eosinophils by expression of HLA-DR and CD14.

### Total viral RNA detection by polymerase chain reaction

RNA was isolated from the nasal wash, throat swabs, and BAL. Samples were inactivated in AVL (Qiagen) and ethanol. Downstream extraction was then performed using the BioSprint™96 One-For-All vet kit (Indical) and Kingfisher Flex platform as per the manufacturer’s instructions. Reverse transcription-quantitative polymerase chain reaction (RT-qPCR) targeting a region of the SARS-CoV-2 nucleocapsid (N) gene was used to determine viral loads and was performed using TaqPath™ 1-Step RT-qPCR Master Mix, CG (Applied Biosystems™), 2019-nCoV CDC RUO Kit (Integrated DNA Technologies) and QuantStudio™ 7 Flex Real-Time PCR System. Sequences of the N1 primers and probe were: 2019-nCoV_N1-forward, 5′ GACCCCAAAATCAGCGAAAT 3′; 2019-nCoV_N1-reverse, 5′ TCTGGTTACTGCCAGTTGAATCTG 3′; 2019-nCoV_N1-probe, 5′ FAM-ACCCCGCATTACGTTTGGTGGACC-BHQ1 3′. The cycling conditions were: 25 °C for 2 m, 50 °C for 15 m, 95 °C for 2 m, followed by 45 cycles of 95 °C for 3 s, 55 °C for 30 s. The quantification standard was in vitro transcribed RNA of the SARS-CoV-2 N ORF (accession number NC_045512.2) with quantification between 1 × 10^1^ and 1 × 10^6^ copies/µl. Positive samples detected below the LOQ were assigned the value of 5 copies/µl, whilst undetected samples were assigned the value of < 2.3 copies/µl, equivalent to the assay’s lower limit of detection.

### Histopathology

#### NHPs

Each animal was assigned a histology number for blinding purposes. The following samples from each animal were fixed in 10% neutral-buffered formalin, processed to paraffin wax and 4 µm thick sections cut and stained with haematoxylin and eosin (H&E); respiratory tract (left cranial and caudal lung lobes), trachea, larynx, tonsil, liver, kidney, spleen, mediastinal lymph node, and small and large intestine. Tissue sections were examined by light microscopy and evaluated subjectively and semi-quantitatively using a scoring system. Pathologists were blinded to treatment and group details and the slides were randomised prior to examination in order to prevent bias (blind evaluation). The slides were reviewed independently by three board-certified veterinary pathologists. For the lung, three sections from each left lung lobe were sampled from different locations: proximal, medial, and distal to the primary lobar bronchus. The scoring system was applied using the following parameters and scores: *Parameters*: bronchial epithelial degeneration/necrosis with the presence of exudates and/or inflammatory cell infiltration. Bronchiolar (primarily terminal) epithelial degeneration/necrosis with the presence of exudates and/or inflammatory cell infiltration. Perivascular inflammatory infiltrates (cuffing). Peribronchiolar inflammatory infiltrates (cuffing). Acute diffuse alveolar damage (necrosis of pneumocytes). Alveolar cellular exudate and oedema and/or fibrin. Alveolar septal inflammatory cells and cellularity. *Scores*: 0 = Normal 1 = Minimal 2 = Mild 3 = Moderate 4 = Severe.

#### Ferrets

A semiquantitative scoring system was developed to compare the severity of the lung lesions for each individual animal and among groups. This scoring system was applied independently to the cranial and caudal lung lobe tissue sections using the following parameters and scores: *Parameters:* bronchial inflammation with the presence of exudates and/or inflammatory cell infiltration. Bronchiolar inflammation with the presence of exudates and/or inflammatory cell infiltration. Perivascular inflammatory infiltrates (cuffing). Infiltration of alveolar walls and spaces by inflammatory cells, mainly mononuclear *Scores:* 0 = None 1 = Minimal 2 = Mild 3 = Moderate 4 = Severe

### Detection of virus by RNAscope

An in-situ hybridisation method used on formalin-fixed, paraffin-embedded tissues was used to identify the SARS-CoV-2 virus in both lung lobes of NHPs. Briefly, tissues were pre-treated with hydrogen peroxide for 10 mins (RT), target retrieval for 15 min (98−101 °C), and protease plus for 30 min (40 °C) (all Advanced Cell Diagnostics). A V-nCoV2019-S probe (Advanced Cell Diagnostics) targeting the S-protein gene was incubated on the tissues for 2 h at 40 °C. Amplification of the signal was carried out following the RNAscope protocol (RNAscope 2.5 HD Detection Reagent—Red) using the RNAscope 2.5 HD red kit (Advanced Cell Diagnostics). Digital image analysis (Nikon NIS-Ar software) was carried out in order to calculate the total area of the lung section positive for viral RNA.

### Statistics and reproducibility

Data in each graph was analysed with a Friedman one-way anova or two-way anova and post hoc test, type of statistical test is noted in the figure legend. Data presented on logarithmic scale were log-transformed prior to analysis. Each individual animal is displayed as an individual point, bars represent medians.

### Reporting summary

Further information on research design is available in the Nature Research Reporting Summary linked to this article.

## Supplementary information

Supplementary Information

Description of Additional Supplementary Files

Supplementary Data 1

Reporting Summary

## Data Availability

The data that support the findings of this study are available within the article and its Supplementary Information files and Supplementary data 1, or are available from the corresponding author upon reasonable request.
