# Supplementary material for: ChAdOx1 nCoV-19 protection against SARS-CoV-2 in rhesus macaque and ferret challenge models
Source: Commun Biol. 2021 Jul 26;4:915. doi: 10.1038/s42003-021-02443-0 (PMC8313674; doi:10.1038/s42003-021-02443-0)
Supplement: Supplementary file 1 — Supplementary Information [file 42003_2021_2443_MOESM1_ESM.pdf]

Supplementary Materials for

ChAdOx1 nCoV-19 protects against SARS-CoV-2 lung damage in rhesus macaques and ferrets

Authors: *T. Lambe[1†], A.J. Spencer[1†], K.M. Thomas[2†], K.E. Gooch[2], S. Thomas[2], A.D. White[2], H.E. Humphries[2], D. Wright[1], S. Belij-Rammerstorfer[1], N. Thakur[3], C. Conceicao[3], R. Watson[2], L. Alden[2], L. Allen[2], M. Aram[2], K.R. Bewley[2], E. Brunt[2], P. Brown[2], B.E. Cavell[2], R. Cobb[2], S.A. Fotheringham[2], C. Gilbride[1], D.J. Harris[2], C.M.K. Ho[2], L. Hunter[2], C.L. Kennard[2], S. Leung[2], V. Lucas[2], D. Ngabo[2], K.A. Ryan[2], H. Sharpe[1], C. Sarfas[2], L. Sibley[2], G.S. Slack[2], M. Ulaszewska[1], N. Wand[2], N. Wiblin[2], F.V. Gleeson[4], D. Bailey[3], S. Sharpe[2], S. Charlton[2], F. J. Salguero[2], M.W. Carroll[2], S.C. Gilbert[1*]*

Correspondence to: sarah.gilbert@ndm.ox.ac.uk

This PDF file includes:

Supplementary text

Figs. S1 to S5

Tables S1 to S7

Supplementary Discussion

CT examination of NHPs following challenge:

NHPs underwent CT scanning on day 5 and 12 post challenge. Fig. S4 shows representative images of pulmonary changes associated with COVID identified five and twelve days after challenge.

ChAdOx1 nCov-19 group: 17Z (male) demonstrated bilateral disease (not shown), ground glass opacities and nodule (yellow arrow) at day 5, was euthanised day 7. Animal 45Z (male) showed normal presentation at day 5, at day 12 presented with unilateral disease with peripheral ground glass opacity (yellow arrow). Animal 13Z (male) showed bilateral disease with subtle ground glass disease present at days 5 and 12, in addition to new middle lobe disease (red arrow) at day 12. Animal 26Z (female) CT scans were normal at day 5, presented with unilateral\ disease at day 12 with very small area of ground glass opacity lower left lobe (yellow arrow). Animals 29Z (female) and 36Z (female) did not show any abnormalities.

PBS control group, animal 22Z male presented with bilateral disease with ground glass opacities in upper lobes (yellow arrow) and lower lobe showing ground glass opacity demonstrating crazy paving, euthanised day 7. Animal 4Z male showed bilateral disease with consolidation and ground glass opacities at day 5, unchanged subtle disease (red arrow), disease improvement (yellow arrow), resolved basal peripheral consolidation (blue arrow) at day 12. Animal 31Z (male), showed normal presentation at day 5, but unilateral disease at day 12 with ground glass opacity in the middle lobe (yellow arrow). Animal 18Z (female), showed bilateral disease with ground glass opacity at days 5 & 12, peripheral consolidation organising pneumonia pattern day 5 (red arrow) resolved by day 12. Animal 53Z (female), showed bilateral disease at day 5 with ground glass opacity in the middle lobe (yellow arrow) and consolidation organising pneumonia pattern (red arrow) in the left lower lobe, disease had resolved by day 12. Animal 24Z (female) had normal presentation at day 5 and was euthanised at day 7.

Overall, there were fewer features of COVID pattern observed in ChAdOx1 nCoV-19 vaccinated animals compared to PBS control animals at day 5, with disease in 2 of 6 vaccinated animals compared to 4 of 6 PBS controls. At day 12 there was equivalent level of COVID pattern features reported in both ChAdOx1 nCoV-19 and PBS animals. The distribution of COVID-abnormalities appeared more restricted in ChAdOx1 nCoV-19 vaccinated animals with ChAdOx1 nCoV-19 showing lower (2 of 2) and peripheral disease (2 of 2), whilst PBS animals showed lower (4 of 4), peripheral (3 of 4) and middle (1 of 4) abnormalities at day 5 post challenge. At day 12 post challenge there was equivalent distribution of disease burden.

NHP histopathology:

Lesions consistent with infection with SARS-CoV-2 were observed in the lungs of animals from both the PBS control and ChAdOx1 nCoV-19 vaccinated groups, no remarkable changes were observed in any of the other tissues examined.

At 7 days post-infection (dpi), varying degrees of pulmonary pathology were observed. The most notable pathology was in control animal 22Z (PBS) which showed multifocal to coalescing areas of interstitial pneumonia, surrounded by areas of unaffected parenchyma. Overall, diffuse alveolar damage (DAD) was a prominent feature in the affected areas, characterised by individual, shrunken, eosinophilic cells in alveolar walls, with pyknotic or karyorrhectic nuclei. In these areas, alveolar spaces were often obliterated by collapse of thickened and damaged alveolar walls which contained mixed inflammatory cells, or had obvious, alveolar type 2 pneumocyte hyperplasia (alveolar epithelialisation). In addition, expanded alveolar spaces filled with fluid and cells comprising fibrillar to homogenous, eosinophilic, proteinaceous fluid (alveolar oedema), admixed with fibrin, polymorph neutrophils (PMNs), enlarged alveolar macrophages and other round cells (possibly detached type 2 pneumocytes). In distal bronchioles and bronchiolo-alveolar junctions, degeneration and sloughing of epithelial cells was present, with areas of attenuation as well as foci of type 2 pneumocyte hyperplasia, representing

regeneration. Hyperplasia and expansion of alveolar macrophages was also evident. Perivascular and peribronchiolar cuffing, mostly composed of mononuclear cells, involved multiple blood vessels and airways. Less severe lesions were observed in the other control animal (29Z), comprising mostly multifocal areas of mild DAD and associated alveolar epithelisation, together with perivascular and peribronchiolar cuffing. In the two vaccinated animals also sacrificed at this timepoint, there were some areas of mild interstitial pneumonia with presence of type 2 pneumocyte hyperplasia, expansion of alveolar macrophages and perivascular/peribronchiolar cuffing; these changes were less severe than those observed in control animal 22Z.
At 13/14dpi, multifocal areas of lung pathology, as described at 7dpi, were noted at reduced severity in three out of the four control animals; in the remaining animal, lesion severity had not reduced. Minimal lesions were also noted in three out of four vaccinated animals; however, in animal 26Z, mild, multifocal interstitial pneumonia (Fig. 3b) and perivascular cuffing, was observed.

At 7dpi, the presence of viral RNA was detected by ISH in both control animals, with a higher staining frequency in animal 22Z (Fig. 3c). Viral RNA was present within pneumocytes and inflammatory cells in the alveolar septae. There was less viral RNA detected in Animal 24Z. Only one vaccinated animal (29Z) had viral RNA in the lung sections; this was associated with lesions within the parenchyma. The remaining animal (17Z), was negative for viral RNA. At 13/14dpi, small amounts of viral RNA were detected in three out of the four control animals, whilst viral RNA was absent all of the vaccinated animals (Fig. 3C). No viral RNA was detectable in extrapulmonary tissues by ISH

Control animals showed similar pulmonary lesions to those observed by our group in previous studies (Salguero et al *Nat Commun* **12,** 1260 (2021)). The pattern of pneumonia was clearly multifocal, with some areas of moderate to severe pathology at 7dpi surrounded by unaffected

parenchyma. At 13/14dpi, the lesions were less severe. Viral RNA was present in areas of pathology and with quantities that correlated with the severity of lesions in both animals at 7dpi and 3/4 animals at 13/14dpi. In general, vaccinated animals showed reduced pathology at 7dpi and 13/14dpi with presence of viral RNA in only one animal at 7dpi and none at 13/14dpi.

Ferret histopathology:

Individual lung histopathology scores are summarised in Table 2. A representative image from the lung and liver from each animal is shown in Fig. 3b and S5.

The most remarkable histological changes were observed in the lung of ferrets. ChAdOx1 nCoV-19 prime only animals did not show any lesion in the lung, apart from minimal inflammatory cell foci within the parenchyma. In ChAdOx1 GFP vaccinated group, two animals showed histopathological changes. Animal 88368 showed mild lesions compatible with the acute bronchiolitis and perivascular/peribronchiolar cuffing, animal 05098 was very similar to ChAdOx1 nCoV-19 vaccinated animals, showing only occasional minimal bronchiolar infiltrates. In the formalin inactivated virus vaccination group, both animals did show more remarkable changes, mild to moderate bronchiolitis (infiltrates within the bronchioles and occasionally bronchi) and inflammatory foci within the parenchyma. Moreover, perivascular cuffing was observed frequently, with the infiltrates being mostly mononuclear cells (occasionally neutrophils and eosinophils were present) (Fig. 4b). The cuffing was also affecting numerous airways.

No remarkable lesions were observed in any other organ except for the liver from all animals. Inflammatory mononuclear infiltrates (mainly periportal) were observed in all animals with varying severity. The most affected animals were 88368 and 09993, showing also occasional mild to multifocal necrosis (Fig. S5c).

RNAScope ISH technique was performed on the lung sections from these 6 animals. Only very few occasional scattered cells were found positive to viral RNA, within the alveolar walls and

not related to the presence of lesions. No differences were observed among groups. It was not possible to carry out digital image analysis, due to the very low presence of positive reaction. RNAScope was performed on the nasal cavities of these 6 animals from the first batch (two each receiving ChAdOx1 nCoV-19 or ChAdOx1 GFP prime only). Viral RNA was found only as small foci of positive cells (epithelial and or sustentacular) within the olfactory and respiratory mucosa in only one animal receiving ChAdOx1 GFP (05098) (data not shown).

At Day 13/14/15 post challenge, the most remarkable histopathological changes were observed in the lung. ChAdOx1 nCoV-19 primed animals showed minimal scattered foci of inflammatory cell infiltration (low grade inflammation), and occasional bronchiolar infiltrates (Fig. S5b). ChAdOx1 GFP primed animals showed minimal to mild perivascular and peribronchiolar infiltrates together with few scattered low grade inflammatory foci (Fig S5b).
RNAScope was performed on lung sections from all the animals, not showing any positive cell in any studied section (data not shown). No remarkable lesions were observed in any other tissue except for the liver that showed a variable degree of multifocal hepatitis, mild to moderate in all the animals (data not shown).

In animals challenged with SARS CoV-2 following prime-boost vaccination, the most remarkable changes were observed in the lung. At 6 dpc, ChAdOx1 nCoV-19 prime boost animals showed minimal scattered foci of inflammatory cell infiltration (low grade inflammation), and occasional bronchiolar infiltrates and perivascular cuffing (Fig. 4b). ChAdOx1 GFP prime boosted animals showed minimal to mild perivascular infiltrates together with few scattered low grade inflammatory foci (Fig. 4b). At 13-14 dpc, animals in both groups showed similar changes with a varying degree of severity, always minimal to mild (Fig. S5b).

Liver sections showed a variable degree of multifocal hepatitis, mild to moderate in all the animals, with some animals showing small foci of necrosis (Fig. S5C). No other changes were observed in the rest of the organs, with the exception of kidney inflammatory mononuclear infiltrates (mild to moderate) in animals #09876 (372/20) and #09848 (374/20) from groups 2 and 3b respectively (data not shown).

Overall prime only animals showed some differences in the lung pathology following challenge with SARS CoV-2, with ChAdOx1 nCoV-19 animals showing only minimal changes. Animals sacrificed 2 weeks after challenge showed a variable degree of inflammatory changes in the lung, within and between groups, from minimal to mild pathology. Animals challenged with SARS CoV-2 following prime-boost vaccination and culled at 6 dpc showed only minimal changes in animals from group compared to minimal to mild pathology observed in animals from the ChAdOx1 GFP group. At 13-14 dpc, animals from both groups showed a variable degree of inflammatory changes in lung, within and between groups, from minimal to mild pathology.
The semiquantitative scoring system was used to discriminate the severity of lesions between animals and groups (Table S6). Even though the number of animals is small, and that lung pathology is not severe in any case, we observed some differences among groups (Fig. 4b). At 6-7dpc, a higher severity was observed in ChAdOx1 GFP and with ChAdOx1 nCoV-19 showing only very minimal changes. At 13-15 dpc, there was some individual variability present and all groups showed a mild degree of pathology. Only ChAdOx1 nCoV-19 vaccinated animals scored a maximum of 1 (minimal) in any parameter studied.
Multifocal hepatitis of varying severity was observed in all ferrets. These lesions are found as a background lesion for this species in many experimental studies, although viral infections, systemic or in the gastrointestinal tract, have been be related to the presence of these periportal inflammatory infiltrates. Due to the variability in severity and the fact that naïve ferrets also showed some degree of hepatitis, the interpretation of this lesion must be taken cautiously. In

two animals from the third batch, a multifocal mild to moderate interstitial cortical nephritis was also observed. This lesion can also be observed in healthy animals, although it has been observed associated to systemic viral infections. Again, the interpretation of these lesions in the kidney must be taken cautiously.

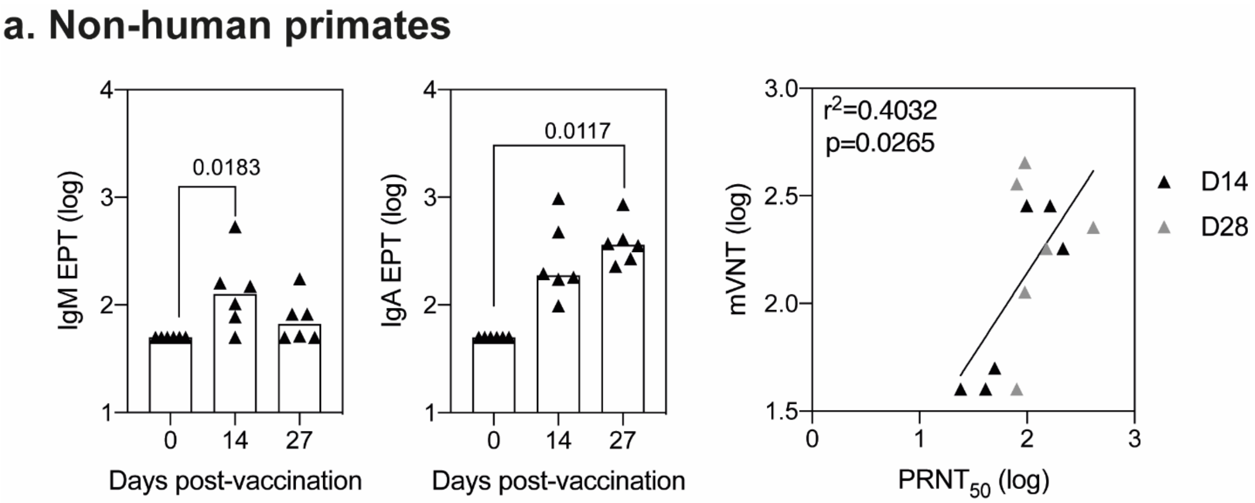

a. Non-human primates
0.0183
IgM EPT (log)
Days post-vaccination
0.0117
IgA EPT (log)
Days post-vaccination
r2=0.4032
p=0.0265
mVNT (log)
PRNT50 (log)
D14
D28

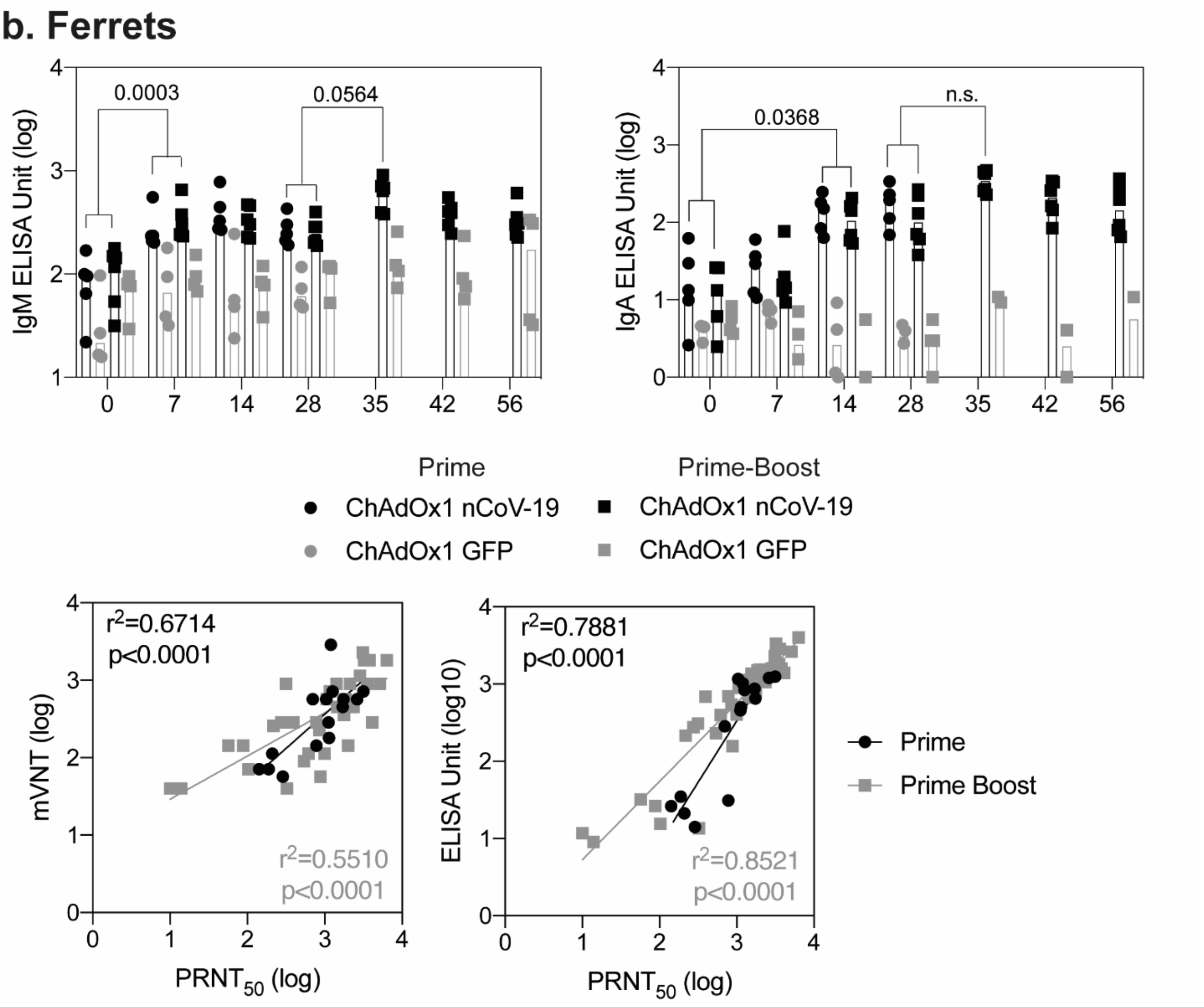

b. Ferrets
0.0003
0.0564
IgM ELISA Unit (log)
0.0368
n.s.
IgA ELISA Unit (log)
Prime
Prime-Boost
ChAdOx1 nCoV-19
ChAdOx1 GFP
ChAdOx1 nCoV-19
ChAdOx1 GFP
r2=0.6714
p<0.0001
r2=0.5510
p<0.0001
mVNT (log)
PRNT50 (log)
r2=0.7881
p<0.0001
r2=0.8521
p<0.0001
ELISA Unit (log10)
PRNT50 (log)
Prime
Prime Boost

Fig S1: Antibody responses in rhesus macaques and ferrets following vaccination with ChAdOx1 nCoV-19

a.) Graphs represent the anti-spike IgM and IgA response as presented by end point titre (EPT) measured in the serum of rhesus macaques following vaccination. Data in each graph was analysed with a one-way anova and post-hoc test with significant differences between timepoints denoted by the line and p value. Graph shows the correlation of neutralisation titres as measured in virus neutralisation or pseduo neutralisation assay of D27 serum samples.

b.) Graphs represent the anti-spike IgM or IgA response presented as an ELISA Unit and correlation between live virus and psudeo-virus neutralisation titres or ELISA Unit at day 28 post vaccination in ferrets vaccinated with ChAdOx1 nCoV-19 (black) or ChAdOx1 GFP. Data in each graph was analysed with a two-way anova and post-hoc test, significant differences from day 0 is noted by bar.

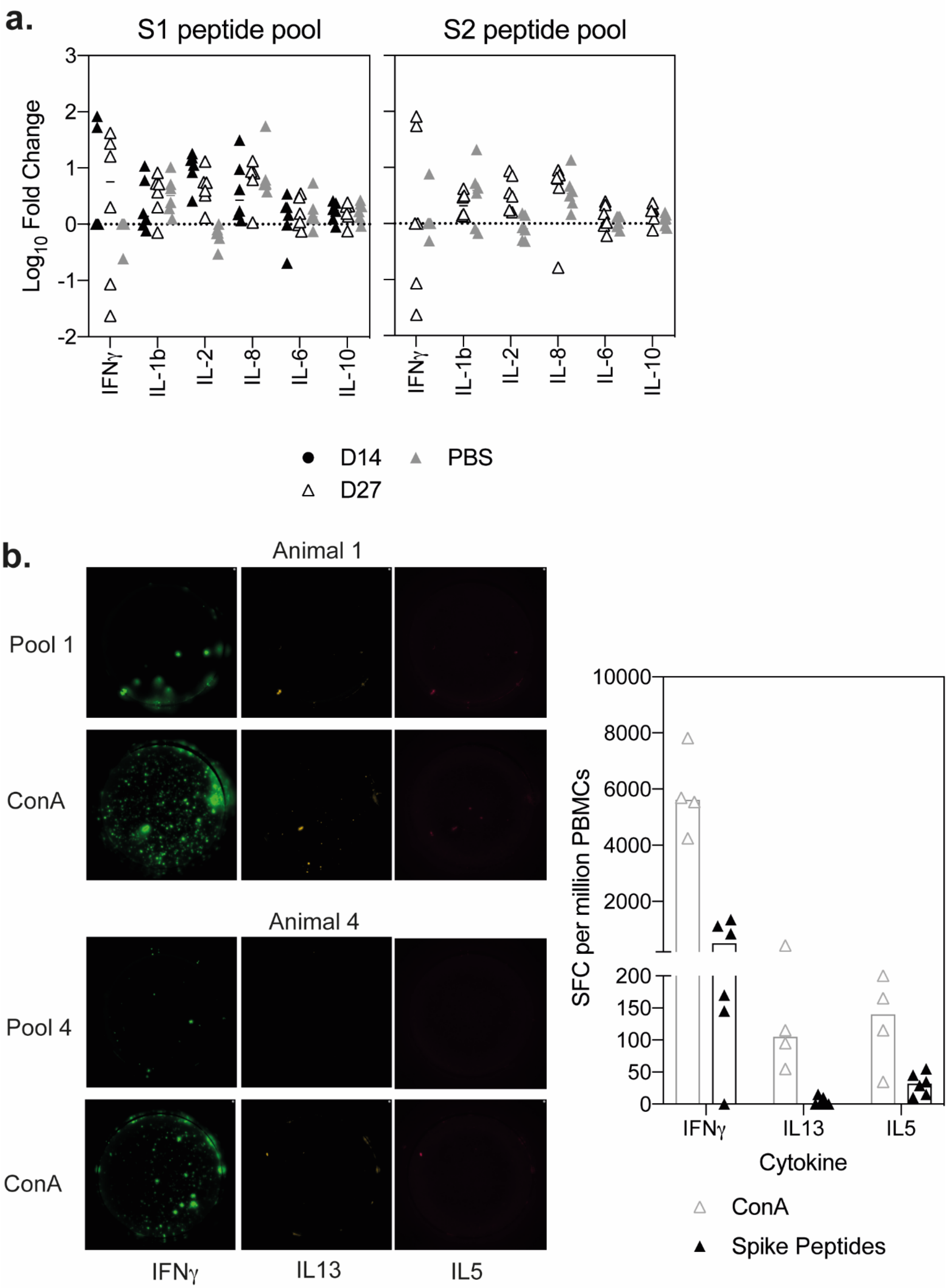
a.
S1 peptide pool
S2 peptide pool
Log10 Fold Change
3
2
1
0
-1
-2
IFNγ
IL-1b
IL-2
IL-8
IL-6
IL-10
D14
PBS
D27
b.
Animal 1
Pool 1
ConA
Animal 4
Pool 4
ConA
IFNγ
IL13
IL5
SFC per million PBMCs
10000
8000
6000
4000
2000
200
150
100
50
0
IFNγ
IL13
IL5
Cytokine
ConA
Spike Peptides

Fig. S2: Spike specific cytokine responses in rhesus macaques following vaccination

a.) PBMCs were stimulated overnight with S1 (pool 1 and 2) and S2 (pool 3 and pool 4) peptides, supernatant collected and levels of cytokines in the supernatant measured. Data is presented as a fold increase in cytokine levels compared to wells containing PMBCs and media.

b.) Detection of IFNγ, IL13 and IL5 was measured by FLUROspot. Data represent the number of cytokine spot forming units per animal when only IFNγ, IL13 or IL5 were detected when PBMCs were stimulated with ConA (grey triangles) or spike peptides (black triangles).

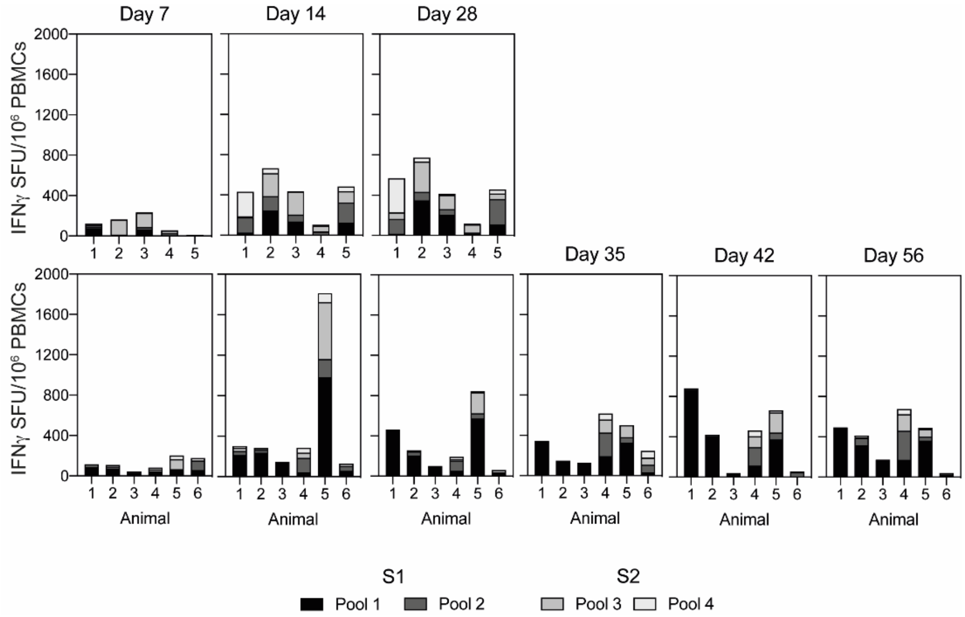

Fig S3: T cell response in ferrets following vaccination

Graphs represent the IFNγ ELISpot response to individual peptide pools in each ferret, following prime (top panel) or prime-boost vaccination with ChAdOx1 nCoV-19.

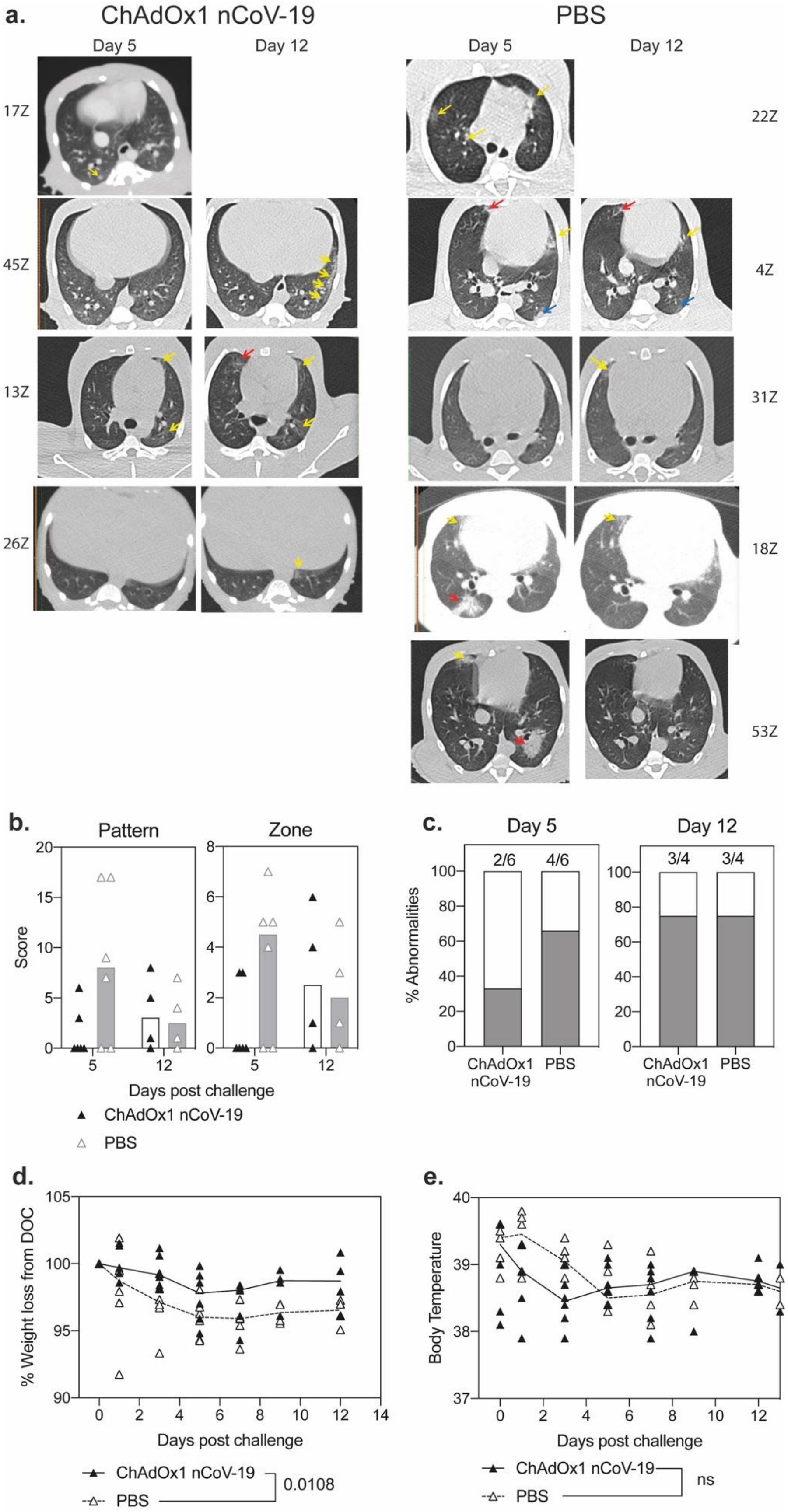
a.
ChAdOx1 nCoV-19
PBS
Day 5
Day 12
Day 5
Day 12
17Z
45Z
13Z
26Z
22Z
4Z
31Z
18Z
53Z
b.
Pattern
Zone
Score
Days post challenge
ChAdOx1 nCoV-19
PBS
c.
Day 5
Day 12
2/6
4/6
3/4
3/4
% Abnormalities
ChAdOx1 nCoV-19
PBS
d.
% Weight loss from DOC
Days post challenge
ChAdOx1 nCoV-19
PBS
0.0108
e.
Body Temperature
Days post challenge
ChAdOx1 nCoV-19
PBS
ns

Figure S4: CT scans of rhesus macaques following challenge with SARS-CoV-2

a.) Representative images of pulmonary changes associated with COVID identified five and twelve days after challenge: ChAdOx1 nCoV-19 group (left panel) 17Z male: bilateral disease (not shown), ground glass opacities and nodule (yellow arrow) at day 5, euthanised day 7; 45Z male: normal at day 5, unilateral disease with peripheral ground glass opacity (yellow arrow) at day 12; 13Z male: bilateral disease with subtle ground glass disease present at days 5 and 12, new middle lobe disease (red arrow) at day 12; 26Z female: normal at day 5, unilateral disease at day 12 with very small area of ground glass opacity lower left lobe (yellow arrow). PBS group, 22Z male: bilateral disease with ground glass opacities in upper lobes (yellow arrow) and lower lobe showing ground glass opacity demonstrating crazy paving, euthanised day 7; 4Z male: bilateral disease with consolidation and ground glass opacities at day 5, unchanged subtle disease (red arrow), disease improvement (yellow arrow), resolved basal peripheral consolidation (blue arrow) at day 12; 31Z male: normal at day 5, unilateral disease at day 12 with ground glass opacity in the middle lobe (yellow arrow); 18Z female: bilateral disease with ground glass opacity at days 5 & 12, peripheral consolidation organising pneumonia pattern day 5 (red arrow) resolved by day 12; 53Z female: Bilateral disease at day 5 with: ground glass opacity in the middle lobe (yellow arrow) and consolidation organising pneumonia pattern (red arrow) in the left lower lobe, disease resolved by day 12. Images from individuals with normal pulmonary lung structure not included. Graphs represent the Pattern and Zone score (**b.**) generated from CT scans taken on Day 5 and Day 12, or proportion of animals with a pulmonary abnormality (Table S1) observed at day 5 or Day 12 (**c.**).

Graphs represent the percentage of weight loss from day of challenge (**d.**) and body temperature (**e.**) in NHP vaccinated with ChAdOx1 nCoV-19 or PBS. Data in each graph was analysed with a 2-way anova to test for an effect of vaccination over time, p values are indicated.

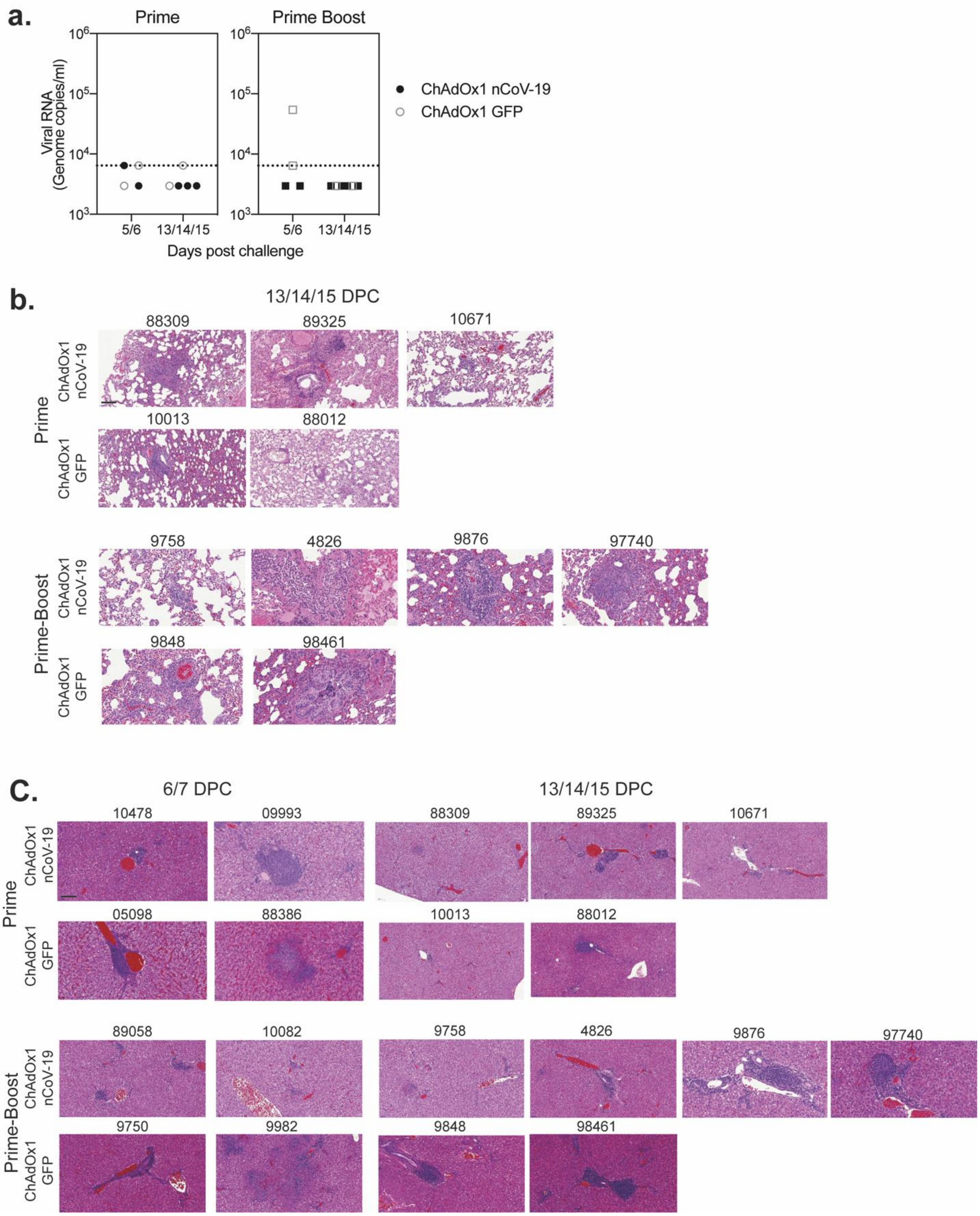

Figure S5: Challenge of ferrets with SARS-CoV-2

a.) Graphs represent quantification of virus RNA by PCR in BALF from ferrets vaccinated with ChAdOx1 nCoV-19 (black closed) or ChAdOx1 GFP controls (grey open) following challenge with SARS-CoV-2. Limit of quantification in the assay was defined as 6430 is indicated as dotted line on the graph. **b.)** Images showed H&E stained sections of ferret lungs of animals sacrificed 2 weeks after challenge with SARS-CoV-2, previously vaccinated with a single dose of vaccine or prime-boost vaccination regimen. Scale bars represent 100μm.

c.) Images showed H&E stained sections of ferret livers of animals sacrificed 1 or 2 weeks after challenge with SARS-CoV-2, previously vaccinated with a single dose of vaccine or prime-boost vaccination regimen. Scale bars represent 100μm.

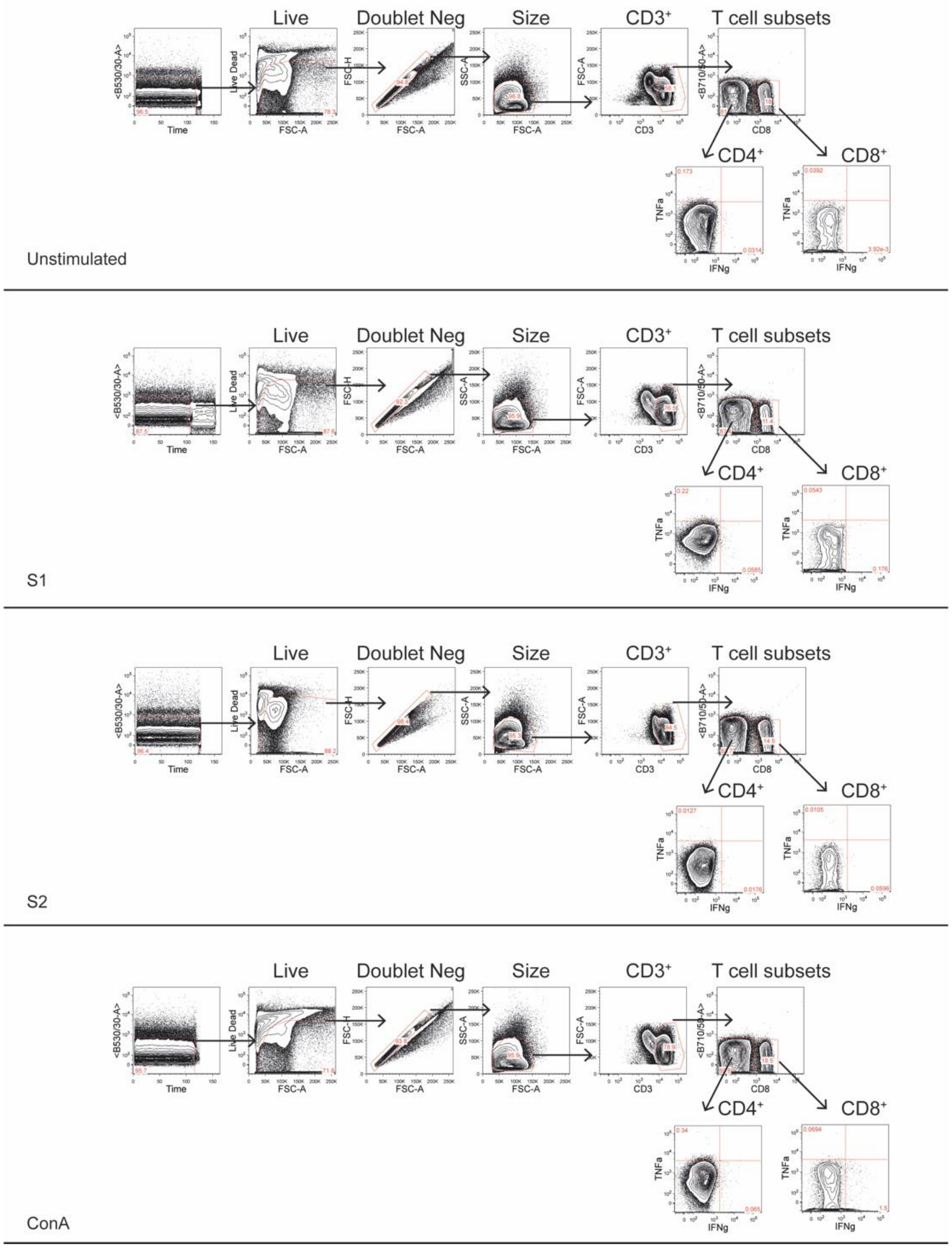

Figure S6: Ferret ICS gating strategy

Ferret PBMCc were stimulated overnight with media, S1 peptide pool, S2 peptide pool or PMA prior to surface and intracellular staining to quantify the frequency of antigen specific IFNγ^+ T cells. Antigen specific T cells were identified by removing debris with a time vs empty channel gate followed by gating on LIVE/DEAD negative, doublet negative (FSC-H vs FSC-A), size (FSC-A vs SSC), CD3$^+$, then CD4$^+$ or CD8$^+$ cells and IFNγ^+.

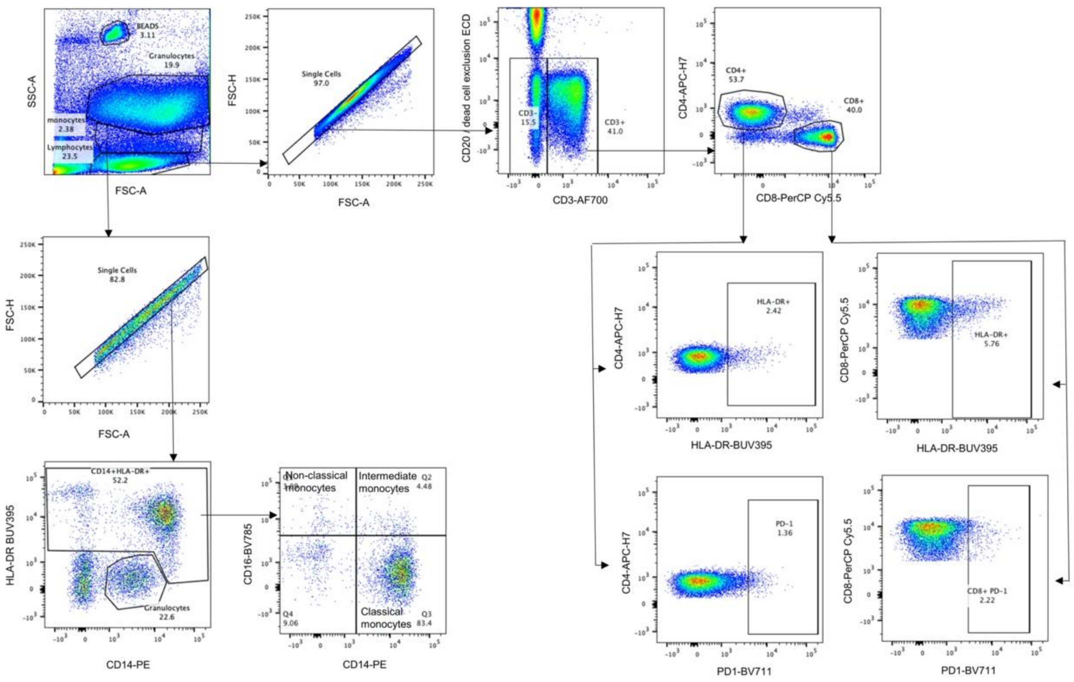

Figure S7: NHP gating strategy

Leukocyte populations were identified using a forward scatter-height (FSC-H) versus side scatter-area (SSC-A) dot plot to identify the lymphocyte, monocyte and granulocyte populations, to which appropriate gating strategies were applied to exclude doublet events and non-viable cells. Lymphocyte sub populations including T-cells, NK-cells, NKT-cells and B-cells were delineated by the expression pattern of CD3, CD20, CD95, CD4, CD8, CD127, CD25, CD16 and the activation and inhibitory markers HLA-DR and PD-1. Classical- and non-classical-monocytes were identified by expression pattern of HLA-DR, CD14 and CD16. Granulocyte populations were delineated into neutrophils and eosinophils by expression of HLA-DR and CD14.

Table S1.

Occurrence of pulmonary abnormalities measured by CT Scan

	Presence of COVID associated abnormalities			
	**ChAdOx1 nCoV-19**		**No vaccine**	
	**Number with abnormalities / number in group**	**% of group with pulmonary abnormalities**	**Number with abnormalities / number in group**	**% of group with pulmonary abnormalities**
**Post challenge**				
**Day 5 or Day12**				
Male	3 of 3	100	3 of 3	100
Female	1 of 3	33	2 of 3	66
Male + Female	4 of 6	66	5 of 6	83
**Day 5**				
Male	2 of 3	66	2 of 3	33
Female	0 of 3	0	2 of 3	33
Male + Female	2 of 6	33	4 of 6	66
**Day 12**				
Male	2 of 2	100	2 of 2	100

Female	1 of 2	50	1 of 2	50
Male + Female	3 of 4	75	3 of 4	75

Table S2.

Occurrence of patterns in pulmonary abnormalities characteristic of COVID

ID	Gender	Status	COVID Pattern (+ / -)											
			Unilateral		Bilateral		GGO		Crazy paving		Peripheral consolidation		Nodule (s)	
			dpc		dpc		dpc		dpc		dpc		dpc	
			5	12	5	12	5	12	5	12	5	12	5	12
17Z	male	ChAdOx1 nCoV-19	-	na	+	na	+	na	-	na	-	na	+	na
13Z	male	ChAdOx1 nCoV-19	+	-	-	+	+	+	-	-	-	-	-	-
45Z	male	ChAdOx1 nCoV-19	-	+	-	-	-	+	-	-	-	-	-	-
29Z	female	ChAdOx1 nCoV-19	-	na	-	na	-	na	-	na	-	na	-	na

26Z	female	ChAdOx1 nCoV-19	-	+	-	-	-	+	-	-	-	-	-	-
36Z	female	ChAdOx1 nCoV-19	-	-	-	-	-	-	-	-	-	-	-	-
22Z	male	PBS	-	na	+	na	+	na	+	na	+	na	-	na
31Z	male	PBS	-	+	-	-	-	+	-	-	-	-	-	-
4Z	male	PBS	-	-	+	+	+	+	-	-	+	-	+	-
18Z	female	PBS	-	-	+	+	+	+	-	-	+	-	-	-
24Z	female	PBS	-	na	-	na	-	na	-	na	-	na	-	na
53Z	female	PBS	-	-	+	-	+	-	-	-	+	-	-	-

ID: identification number; dpc: days post challenge; GGO: ground glass opacification; na: not applicable

Table S3

Distribution of pulmonary abnormalities characteristic of COVID

ID	Gender	Status	Distribution (+ / -)											
			Upper		Middle		Lower		Random		Central 2/3		Peripheral 2/3	
			dpc		dpc		dpc		dpc		dpc		dpc	
			5	12	5	12		12	5	12	5	12	5	12
17Z	male	ChAdOx1 nCoV-19	-	na	+	na	+	na	-	na	-	na	+	na
13Z	male	ChAdOx1 nCoV-19	+	+	-	+	+	+	-	-	-	-	+	+
45Z	male	ChAdOx1 nCoV-19	-	+	-	+	-	+	-	-	-	-	-	+
29Z	female	ChAdOx1 nCoV-19	-	na	-	na	-	na	-	na	-	na	-	na
26Z	female	ChAdOx1 nCoV-19	-	-	-	-	-	+	-	-	-	-	-	+
36Z	female	ChAdOx1 nCoV-19	-	-	-	-	-	-	-	-	-	-	-	-
22Z	male	PBS	+	na	+	na	+	na	+	na	-	na	-	na
31Z	male	PBS	-	-	-	+	-	-	-	-	-	-	-	+

4Z	male	PBS	-	-	+	+	+	+	+	-	-	-	-	+
18Z	female	PBS	-	-	-	-	+	+	-	-	-	-	+	+
24Z	female	PBS	-	na	-	na	-	na	-	na	-	na	-	na
53Z	female	PBS	-	-	+	-	+	-	-	-	-	-	+	-

ID: identification number; dpc: days post challenge; na: not applicable

Table S4

Pulmonary Disease burden measured using a quantitative score system

ID	Gender	Status	COVID pattern parameter scores								Zone Score		Total score	
			GGO		Consoli-dation score		Nodule score		Total					
			dpc		dpc		dpc		dpc		dpc		dpc	
			5	12	5	12	5	12	5	12	5	12	5	12
17Z	male	ChAdOx1 nCoV-19	5	na	0	na	1	na	6	na	3	na	9	na
13Z	male	ChAdOx1 nCoV-19	3	5	0	0	0	0	3	5	3	6	6	11
45Z	male	ChAdOx1 nCoV-19	0	8	0	0	0	0	0	8	0	4	0	12
29Z	female	ChAdOx1 nCov-19	0	na	0	na	0	na	0	na	0	na	0	na
26Z	female	ChAdOx1 nCoV-19	0	1	0	0	0	0	0	1	0	1	0	2
36Z	female	ChAdOx1 nCoV-19	0	0	0	0	0	0	0	0	0	0	0	0
22Z	male	PBS	9	na	8	na	0	na	17	na	7	na	24	na

31Z	male	PBS	0	1	0	0	0	0	0	1	0	1	0	2
4Z	male	PBS	4	7	4	0	1	0	9	7	5	5	14	12
18Z	female	PBS	1	4	6	0	0	0	7	4	4	3	11	7
24Z	female	PBS	0	na	0	na	0	na	0	na	0	na	0	na
53Z	female	PBS	1	0	16	0	0	0	17	0	5	0	22	0

ID: identification number; dpc: days post challenge; GGO: ground glass opacification; na: not applicable.

Table S5. Rhesus Macaques pulmonary histopathology scoring system

Lesion	Score 0 (normal)	Score 1 (minimal)	Score 2 (mild)	Score 3 (moderate)	Score 4 (severe)
***Bronchial* epithelial degeneration/ necrosis with presence of exudates and/or inflammatory cell infiltration**	None	Occasional (1 or 2) bronchi affected.	Present in multiple airways; **up to 25% of bronchi affected**	Present in multiple airways; **between 26-50% of bronchi affected**	Present in multiple airways; **over 50% of bronchi affected**
***Bronchiolar* (primarily terminal) epithelial degeneration/**	None	Occasional (1 or 2) bronchioli affected	Present in multiple airways; **up to 25% of**	Present in multiple airways; **between 26-50% of**	Present in multiple airways; **over 50% of**

**necrosis with presence of exudates and/or inflammatory cell infiltration**			**bronchioli affected**	**bronchioli affected**	**bronchioli affected**
***Perivascular* inflammatory infiltrates (cuffing)**	None	**Occasional** incomplete, or loosely formed cuffs	**Numerous** cuffs; predominantly **incomplete** and **loosely formed** with **lesser well-formed complete cuffs**	**Numerous** cuffs; approximately **half or more** well-formed, and may have **few broad, dense cuffs**	**Numerous** cuffs; **predominantly well-formed** with **numerous broad, dense cuffs**

***Peribronchiolar* inflammatory infiltrates (cuffing)**	None	**Occasional** incomplete, or loosely formed cuffs	**Numerous** cuffs; predominantly **incomplete** and **loosely formed** with **lesser well-formed complete cuffs**	**Numerous** cuffs; approximately **half or more** well-formed, and may have **few broad, dense cuffs**	**Numerous** cuffs; **predominantly well-formed** with **numerous broad, dense cuffs**
**Acute diffuse alveolar damage (necrosis of pneumocytes)**	None	Small numbers of foci; **up to 5% of slide affected**	Multiple foci; **between 6-25% of the slide affected**	Increased numbers of foci; between **26-50% of the slide affected**	Numerous foci; over **50% of the slide affected**
**Alveolar cellular exudate and**	None (alveolar macrophages at	**Occasional** alveoli; **up to**	Confluent alveoli; between **6-**	Confluent alveoli; between **26-**	Confluent alveoli; **affecting over**

**oedema and/or fibrin**	physiological levels)	**5% of slide affected**	**25% of the slide affected**	**50% of the slide affected**	**50% of the slide**
**Alveolar septal inflammatory cells and cellularity**	Normal septae; typically 1-2 (occasionally 3) nucleated cells wide; absence of inflammatory cells	Thickening of the alveolar walls by inflammatory cells; **up to 5% of the slide affected**	Thickening of the alveolar walls by inflammatory cells; **between 6-25% of the slide affected**	Thickening of the alveolar walls by inflammatory cells; **between 26-50% of the slide affected**	Thickening of the alveolar walls by inflammatory cells; **over 50% of the slide affected**

Table S6. Histopathological scores for each individual ferret

					Cranial lung lobe Histopath Score				Caudal lung lobe Histopath Score				Total
Group	Lab ID	Animal ID	Histo ref	dpc	Bronchial	Bronchiolar	PV cuffing	Alveoli	Bronchial	Bronchiolar	PV cuffing	Alveoli	Score
1 ChAdOx1-nCoV-19 prime only	1-1	10478	229/20	6dpc	0	0	0	1	0	0	0	1	2
	1-2	9993	230/20	6dpc	0	0	0	1	0	0	0	1	2
	1-3	88309	292/20	13dpc	0	0	0	1	1	0	1	1	4
	1-4	89325	293/20	13dpc	0	1	0	1	1	0	1	1	5
	1-5	10671	294/20	14dpc	0	0	0	1	0	0	1	1	3
	1-6	9833	134/20	PM									
2 ChAdOx1-nCoV-19 prime boost	2-1	89058	366/20	6dpc	0	1	1	0	0	1	0	0	3
	2-2	10082	367/20	6dpc	0	1	0	1	0	1	0	0	3
	2-3	9758	370/20	13dpc	0	0	0	1	1	0	0	1	3
	2-4	4826	371/20	13dpc	0	2	0	0	1	2	1	0	6
	2-5	9876	372/20	14dpc	0	0	0	1	0	0	1	2	4
	2-6	87740	373/20	14dpc	0	0	1	1	0	1	1	1	5
3a	3a-1	5098	231/20	6dpc	0	1	0	1	0	0	0	1	3
	3a-2	88368	232/20	6dpc	0	2	1	1	0	1	1	1	7

ChAdOx1-GFP prime only	3a-3	10013	295/20	13dpc	0	0	1	1	0	0	1	1	4
	3a-4	88012	296/20	14dpc	0	1	1	1	0	0	2	1	6
3b ChAdOx1-GFP prime boost	3b-1	9750	368/20	6dpc	1	2	1	0	0	2	1	1	8
	3b-2	9982	369/20	6dpc	1	2	1	0	1	2	1	0	8
	3b-3	9848	374/20	13dpc	1	1	2	1	0	0	1	1	7
	3b-4	89461	375/20	14dpc	1	1	1	1	0	2	1	1	8

Table S7. SARs CoV-2 overlapping peptide sequences

S1				S2			
Pool 1		Pool 2		Pool 3		Pool 4	
#	Sequence	#	Sequence	#	Sequence	#	Sequence
1	MFVFLVLLPLVSSQC	78	EKGIYQTSNFRVQPT	168	GICASYQTQTNSPRR	242	QLSSNFGAISSVLND
2	LVLLPLVSSQCVNLT	79	YQTSNFRVQPTESIV	169	SYQTQTNSPRRARSV	243	NFGAISSVLNDILSR
3	PLVSSQCVNLTTRTQ	80	NFRVQPTESIVRFPN	170	QTNSPRRARSVASQS	244	ISSVLNDILSRLDKV
4	SQCVNLTTRTQLPPA	81	QPTESIVRFPNITNL	171	PRRARSVASQSIIAY	245	LNDILSRLDKVEAEV
5	NLTTRTQLPPAYTNS	82	SIVRFPNITNLCPFG	172	RSVASQSIIAYTMSL	246	LSRLDKVEAEVQIDR
6	RTQLPPAYTNSFTRG	83	FPNITNLCPFGEVFN	173	SQSIIAYTMSLGAEN	247	DKVEAEVQIDRLITG
7	PPAYTNSFTRGVYYP	84	TNLCPFGEVFNATRF	174	IAYTMSLGAENSVAY	248	AEVQIDRLITGRLQS
8	TNSFTRGVYYPDKVF	85	PFGEVFNATRFASVY	175	MSLGAENSVAYSNNS	249	IDRLITGRLQSLQTY
9	TRGVYYPDKVFRSSV	86	VFNATRFASVYAWNR	176	AENSVAYSNNSIAIP	250	ITGRLQSLQTYVTQQ
10	YYPDKVFRSSVLHST	87	TRFASVYAWNRKRIS	177	VAYSNNSIAIPTNFT	251	LQSLQTYVTQQLIRA
11	KVFRSSVLHSTQDLF	88	SVYAWNRKRISNCVA	178	NNSIAIPTNFTISVT	252	QTYVTQQLIRAAEIR
12	SSVLHSTQDLFLPFF	89	WNRKRISNCVADYSV	179	AIPTNFTISVTTEIL	253	TQQLIRAAEIRASAN
13	HSTQDLFLPFFSNVT	90	RISNCVADYSVLYNS	180	NFTISVTTEILPVSM	254	IRAAEIRASANLAAT
14	DLFLPFFSNVTWFHA	91	CVADYSVLYNSASFS	181	SVTTEILPVSMTKTS	255	EIRASANLAATKMSE
15	PFFSNVTWFHAIHVS	92	YSVLYNSASFSTFKC	182	EILPVSMTKTSVDCT	256	SANLAATKMSECVLG
16	NVTWFHAIHVSGTNG	93	YNSASFSTFKCYGVS	183	VSMTKTSVDCTMYIC	257	AATKMSECVLGQSKR
17	FHAIHVSGTNGTKRF	94	SFSTFKCYGVSPTKL	184	KTSVDCTMYICGDST	258	MSECVLGQSKRVDFC
18	HVSGTNGTKRFDNPV	95	FKCYGVSPTKLNDLC	185	DCTMYICGDSTECSN	259	VLGQSKRVDFCGKGY
19	TNGTKRFDNPVLPFN	96	GVSPTKLNDLCFTNV	186	YICGDSTECSNLLLQ	260	SKRVDFCGKGYHLMS
20	KRFDNPVLPFNDGVY	97	TKLNDLCFTNVYADS	187	DSTECSNLLLQYGSF	261	DFCGKGYHLMSFPQS
21	NPVLPFNDGVYFAST	98	DLCFTNVYADSFVIR	188	CSNLLLQYGSFCTQL	262	KGYHLMSFPQSAPHG
22	PFNDGVYFASTEKSN	99	TNVYADSFVIRGDEV	189	LLQYGSFCTQLNRAL	263	LMSFPQSAPHGVVFL
23	GVYFASTEKSNIIRG	100	ADSFVIRGDEVRQIA	190	GSFCTQLNRALTGIA	264	PQSAPHGVVFLHVTY
24	ASTEKSNIIRGWIFG	101	VIRGDEVRQIAPGQT	191	TQLNRALTGIAVEQD	265	PHGVVFLHVTYVPAQ
25	KSNIIRGWIFGTTLD	102	DEVRQIAPGQTGKIA	192	RALTGIAVEQDKNTQ	266	VFLHVTYVPAQEKNF
26	IRGWIFGTTLDSKTQ	103	QIAPGQTGKIADYNY	193	GIAVEQDKNTQEVFA	267	VTYVPAQEKNFTTAP
27	IFGTTLDSKTQSLLI	104	GQTGKIADYNYKLPD	194	EQDKNTQEVFAQVKQ	268	PAQEKNFTTAPAICH
28	TLDSKTQSLLIVNNA	105	KIADYNYKLPDDFTG	195	NTQEVFAQVKQIYKT	269	KNFTTAPAICHDGKA
29	KTQSLLIVNNATNVV	106	YNYKLPDDFTGCVIA	196	VFAQVKQIYKTPPIK	270	TAPAICHDGKAHFPR
30	LLIVNNATNVVIKVC	107	LPDDFTGCVIAWNSN	197	VKQIYKTPPIKDFGG	271	ICHDGKAHFPREGVF
31	NNATNVVIKVCEFQF	108	FTGCVIAWNSNNLDS	198	YKTPPIKDFGGFNFS	272	GKAHFPREGVFVSNG
32	NVVIKVCEFQFCNDP	109	VIAWNSNNLDSKVGG	199	PIKDFGGFNFSQILP	273	FPREGVFVSNGTHWF
33	KVCEFQFCNDPFLGV	110	NSNNLDSKVGGNYNY	200	FGGFNFSQILPDPSK	274	GVFVSNGTHWFVTQR
34	FQFCNDPFLGVYYHK	111	LDSKVGGNYNYLYRL	201	NFSQILPDPSKPSKR	275	SNGTHWFVTQRNFYE
35	NDPFLGVYYHKNNKS	112	VGGNYNYLYRLFRKS	202	ILPDPSKPSKRSFIE	276	HWFVTQRNFYEPQII
36	LGVYYHKNNKSWMES	113	YNYLYRLFRKSNLKP	203	PSKPSKRSFIEDLLF	277	TQRNFYEPQIITTDN
37	YHKNNKSWMESEFRV	114	YRLFRKSNLKPFERD	204	SKRSFIEDLLFNKVT	278	FYEPQIITTDNTFVS
38	NKSWMESEFRVYSSA	115	RKSNLKPFERDISTE	205	FIEDLLFNKVTLADA	279	QIITTDNTFVSGNCD
39	MESEFRVYSSANNCT	116	LKPFERDISTEIYQA	206	LLFNKVTLADAGFIK	280	TDNTFVSGNCDVVIG
40	FRVYSSANNCTFEYV	117	ERDISTEIYQAGSTP	207	KVTLADAGFIKQYGD	281	FVSGNCDVVIGIVNN
41	SSANNCTFEYVSQPF	118	STEIYQAGSTPCNGV	208	ADAGFIKQYGDCLGD	282	NCDVVIGIVNNTVYD
42	NCTFEYVSQPFLMDL	119	YQAGSTPCNGVEGFN	209	FIKQYGDCLGDIAAR	283	VIGIVNNTVYDPLQP
43	EYVSQPFLMDLEGKQ	120	STPCNGVEGFNCYFP	210	YGDCLGDIAARDLIC	284	VNNTVYDPLQPELDS
44	QPFLMDLEGKQGNFK	121	NGVEGFNCYFPLQSY	211	LGDIAARDLICAQKF	285	VYDPLQPELDSFKEE
45	MDLEGKQGNFKNLRE	122	GFNCYFPLQSYGFQP	212	AARDLICAQKFNGLT	286	LQPELDSFKEELDKY
46	GKQGNFKNLREFVFK	123	YFPLQSYGFQPTNGV	213	LICAQKFNGLTVLPP	287	LDSFKEELDKYFKNH
47	NFKNLREFVFKNIDG	124	QSYGFQPTNGVGYQP	214	QKFNGLTVLPPLLTD	288	KEELDKYFKNHTSPD
48	LREFVFKNIDGYFKI	125	FQPTNGVGYQPYRVV	215	GLTVLPPLLTDEMIA	289	DKYFKNHTSPDVDLG
49	VFKNIDGYFKIYSKH	126	NGVGYQPYRVVVLSF	216	LPPLLTDEMIAQYTS	290	KNHTSPDVDLGDISG
50	IDGYFKIYSKHTPIN	127	YQPYRVVVLSFELLH	217	LTDEMIAQYTSALLA	291	SPDVDLGDISGINAS
51	FKIYSKHTPINLVRD	128	RVVVLSFELLHAPAT	218	MIAQYTSALLAGTIT	292	DLGDISGINASVVNI
52	SKHTPINLVRDLPQG	129	LSFELLHAPATVCGP	219	YTSALLAGTITSGWT	293	ISGINASVVNIQKEI
53	PINLVRDLPQGFSAL	130	LLHAPATVCGPKKST	220	LLAGTITSGWTFGAG	294	NASVVNIQKEIDRLN
54	VRDLPQGFSALEPLV	131	PATVCGPKKSTNLVK	221	TITSGWTFGAGAALQ	295	VNIQKEIDRLNEVAK
55	PQGFSALEPLVDLPI	132	CGPKKSTNLVKNKCV	222	GWTFGAGAALQIPFA	296	KEIDRLNEVAKNLNE
56	SALEPLVDLPIGINI	133	KSTNLVKNKCVNFNF	223	GAGAALQIPFAMQMA	297	RLNEVAKNLNESLID
57	PLVDLPIGINITRFQ	134	LVKNKCVNFNFNGLT	224	ALQIPFAMQMAYRFN	298	VAKNLNESLIDLQEL
58	LPIGINITRFQTLLA	135	KCVNFNFNGLTGTGV	225	PFAMQMAYRFNGIGV	299	LNESLIDLQELGKYE
59	INITRFQTLLALHRS	136	FNFNGLTGTGVLTES	226	QMAYRFNGIGVTQNV	300	LIDLQELGKYEQYIK
60	RFQTLLALHRSYLTP	137	GLTGTGVLTESNKKF	227	RFNGIGVTQNVLYEN	301	QELGKYEQYIKWPWY
61	LLALHRSYLTPGDSS	138	TGVLTESNKKFLPFQ	228	IGVTQNVLYENQKLI	302	KYEQYIKWPWYIWLG
62	HRSYLTPGDSSSGWT	139	TESNKKFLPFQQFGR	229	QNVLYENQKLIANQF	303	YIKWPWYIWLGFIAG
63	LTPGDSSSGWTAGAA	140	KKFLPFQQFGRDIAD	230	YENQKLIANQFNSAI	304	PWYIWLGFIAGLIAI
64	DSSSGWTAGAAAYYV	141	PFQQFGRDIADTTDA	231	KLIANQFNSAIGKIQ	305	WLGFIAGLIAIVMVT
65	GWTAGAAAYYVGYLQ	142	FGRDIADTTDAVRDP	232	NQFNSAIGKIQDSLS	306	IAGLIAIVMVTIMLC
66	GAAAYYVGYLQPRTF	143	IADTTDAVRDPQTLE	233	SAIGKIQDSLSSTAS	307	IAIVMVTIMLCCMTS
67	YYVGYLQPRTFLLKY	144	TDAVRDPQTLEILDI	234	KIQDSLSSTASALGK	308	MVTIMLCCMTSCCSC
68	YLQPRTFLLKYNENG	145	RDPQTLEILDITPCS	235	SLSSTASALGKLQDV	309	MLCCMTSCCSCLKGC

69	RTFLLKYNENGTITD	146	TLEILDITPCSFGGV	236	TASALGKLQDVVNQN	310	MTSCCSCLKGCCSCG
70	LKYNENGTITDAVDC	147	LDITPCSFGGVSVIT	237	LGKLQDVVNQNAQAL	311	CSCLKGCCSCGSCCK
71	ENGTITDAVDCALDP	148	PCSFGGVSVITPGTN	238	QDVVNQNAQALNTLV	312	KGCCSCGSCCKFDED
72	ITDAVDCALDPLSET	149	GGVSVITPGTNTSNQ	239	NQNAQALNTLVKQLS	313	SCGSCCKFDEDDSEP
73	VDCALDPLSETKCTL	150	VITPGTNTSNQVAVL	240	QALNTLVKQLSSNFG	314	CCKFDEDDSEPVLKG
74	LDPLSETKCTLKSFT	151	GTNTSNQVAVLYQDV	241	TLVKQLSSNFGAISS	315	DEDDSEPVLKGVKLH
75	SETKCTLKSFTVEKG	152	SNQVAVLYQDVNCTE			316	DDSEPVLKGVKLHYT
76	CTLKSFTVEKGIYQT	153	AVLYQDVNCTEVPVA				
77	SFTVEKGIYQTSNFR	154	QDVNCTEVPVAIHAD				
		155	CTEVPVAIHADQLTP				
		156	PVAIHADQLTPTWRV				
		157	HADQLTPTWRVYSTG				
		158	LTPTWRVYSTGSNVF				
		159	WRVYSTGSNVFQTRA				
		160	STGSNVFQTRAGCLI				
		161	NVFQTRAGCLIGAEH				
		162	TRAGCLIGAEHVNNS				
		163	CLIGAEHVNNSYECD				
		164	AEHVNNSYECDIPIG				
		165	NNSYECDIPIGAGIC				
		166	ECDIPIGAGICASYQ				
		167	PIGAGICASYQTQTN				
