# Supplementary material for: ChAdOx1 nCoV-19 protection against SARS-CoV-2 in rhesus macaque and ferret challenge models
Source: Commun Biol. 2021 Jul 26;4:915. doi: 10.1038/s42003-021-02443-0 (PMC8313674; doi:10.1038/s42003-021-02443-0)
Supplement: Supplementary file 2 — Description of Additional Supplementary Files [file 42003_2021_2443_MOESM2_ESM.pdf]

Description of Additional Supplementary Files

File Name: Supplementary Data 1
Description: Source Data
