# Supplementary material for: ChAdOx1 nCoV-19 protection against SARS-CoV-2 in rhesus macaque and ferret challenge models
Source: Commun Biol. 2021 Jul 26;4:915. doi: 10.1038/s42003-021-02443-0 (PMC8313674; doi:10.1038/s42003-021-02443-0)
Supplement: Supplementary file 4 — Reporting Summary [file 42003_2021_2443_MOESM4_ESM.pdf]

nature research

Corresponding author(s): Sarah Gilbert

Last updated by author(s): 19th June 2021

Reporting Summary

Nature Research wishes to improve the reproducibility of the work that we publish. This form provides structure for consistency and transparency in reporting. For further information on Nature Research policies, see our Editorial Policies and the Editorial Policy Checklist.

Statistics

For all statistical analyses, confirm that the following items are present in the figure legend, table legend, main text, or Methods section.

n/a	Confirmed	
☐	☒	The exact sample size (n) for each experimental group/condition, given as a discrete number and unit of measurement
☐	☒	A statement on whether measurements were taken from distinct samples or whether the same sample was measured repeatedly
☐	☒	The statistical test(s) used AND whether they are one- or two-sided<br>*Only common tests should be described solely by name; describe more complex techniques in the Methods section.*
☒	☐	A description of all covariates tested
☐	☒	A description of any assumptions or corrections, such as tests of normality and adjustment for multiple comparisons
☐	☒	A full description of the statistical parameters including central tendency (e.g. means) or other basic estimates (e.g. regression coefficient) AND variation (e.g. standard deviation) or associated estimates of uncertainty (e.g. confidence intervals)
☐	☒	For null hypothesis testing, the test statistic (e.g. F, t, r) with confidence intervals, effect sizes, degrees of freedom and P value noted<br>*Give P values as exact values whenever suitable.*
☒	☐	For Bayesian analysis, information on the choice of priors and Markov chain Monte Carlo settings
☒	☐	For hierarchical and complex designs, identification of the appropriate level for tests and full reporting of outcomes
☒	☐	Estimates of effect sizes (e.g. Cohen's d, Pearson's r), indicating how they were calculated

Our web collection on statistics for biologists contains articles on many of the points above.

Software and code

Policy information about availability of computer code

Data collection	BD FACs Diva for collection of flow cytometry data. AID ELISpot or MabTech SpotReader for counting ELISpots.
Data analysis	GraphPad Prism v8.1.2; FlowJo v9; FlowJo X, AID ELISpot V5, AID ELISpot v, Mabtech SpotReader, SOFTmax PRO software, Nikon NIS-Ar

For manuscripts utilizing custom algorithms or software that are central to the research but not yet described in published literature, software must be made available to editors and reviewers. We strongly encourage code deposition in a community repository (e.g. GitHub). See the Nature Research guidelines for submitting code & software for further information.

Data

Policy information about availability of data

All manuscripts must include a data availability statement. This statement should provide the following information, where applicable:

- Accession codes, unique identifiers, or web links for publicly available datasets
- A list of figures that have associated raw data
- A description of any restrictions on data availability

The data that support the findings of this study are available from the corresponding authors upon reasonable request.

Field-specific reporting

Life sciences study design

All studies must disclose on these points even when the disclosure is negative.

Sample size	ChAdOx1 nCoV-19 vaccinated groups
Data exclusions	Ferret 1-6 was steadily losing weight throughout the initial period of the study since arrival (5 days prior to vaccination). The animal was euthanised on welfare grounds on study day 14 after being scored as lethargic, with gait changes and reduced activity score. As the weight loss was noted from arrival it is not likely related to the vaccination. Data from this animal was excluded from the study.
Replication	Data were not replicated.
Randomization	Animals were randomly allocated to treatment groups.
Blinding	Samples were blinded to the pathologist and for pseduo-neutralisation assays.

Reporting for specific materials, systems and methods

We require information from authors about some types of materials, experimental systems and methods used in many studies. Here, indicate whether each material, system or method listed is relevant to your study. If you are not sure if a list item applies to your research, read the appropriate section before selecting a response.

Materials & experimental systems

n/a	Involved in the study
☐	☒ Antibodies
☐	☒ Eukaryotic cell lines
☒	☐ Palaeontology and archaeology
☐	☒ Animals and other organisms
☒	☐ Human research participants
☒	☐ Clinical data
☒	☐ Dual use research of concern

Methods

n/a	Involved in the study
☒	☐ ChIP-seq
☐	☒ Flow cytometry
☒	☐ MRI-based neuroimaging

Antibodies

Antibodies used	For ferret flow cytometry staining: anti-ferret CD3 Alexa 405 (Clone PC3/188A) (Santa Cruz Biotechnology), anti-human CD8 APCCy7 (Clone OKT8) (Thermofisher), Thermofisher), anti-bovine IFNg PE (Clone CC302) (Abserotec), anti-mouse TNFa A647 (Clone MP6-XT22).<br>For NHP flow cytometry staining: anti-CD3-AF700, anti-CD4-APC-H7, anti-CD8-PerCP-Cy5.5, anti-CD95-Pe-Cy7, anti-CD14-PE, anti-HLA-DR-BUV395, anti-CD25-FITC (all from BD Biosciences, Oxford, UK); anti-γδ-TCR-BV421, anti-CD16-BV786, anti-CD20-PE-Dazzle, anti-CD279 (PD1) (all from BioLegend).
Validation	mAb clones were selected based on published data confirming binding and for use with NHP or ferret samples.

Eukaryotic cell lines

Policy information about cell lines

Cell line source(s)	*State the source of each cell line used.*
Authentication	*Describe the authentication procedures for each cell line used OR declare that none of the cell lines used were authenticated.*
Mycoplasma contamination	*Confirm that all cell lines tested negative for mycoplasma contamination OR describe the results of the testing for mycoplasma contamination OR declare that the cell lines were not tested for mycoplasma contamination.*
Commonly misidentified lines<br>(See ICLAC register)	*Name any commonly misidentified cell lines used in the study and provide a rationale for their use.*

Animals and other organisms

Policy information about studies involving animals; ARRIVE guidelines recommended for reporting animal research

Laboratory animals	Twelve rhesus macaques of Indian origin (Macaca mulatta) were used in this study. Study groups comprised three males and three females and all were adults aged 4 years and weighing between 4.30 and 8.24kg at time of challenge.<br>Twenty-eight healthy, female ferrets (Mustela putorius furo) aged 5-7 months were obtained from a UK Home Office accredited supplier (Highgate Farm, UK). The mean weight at the time of challenge was 973 g/ferret (range 825 to 1129g).

Wild animals	no wild animals were used
Field-collected samples	This study did not involve field collected samples.
Ethics oversight	All experimental work was conducted under the authority of a UK Home Office approved project license (NHPs:PDC57C033, Ferrets:PDC57C033) that had been subject to local ethical review at PHE Porton Down by the Animal Welfare and Ethical Review Body (AWERB) and approved as required by the Home Office Animals (Scientific Procedures) Act 1986.

Note that full information on the approval of the study protocol must also be provided in the manuscript.

Flow Cytometry

Plots

Confirm that:

☒ The axis labels state the marker and fluorochrome used (e.g. CD4-FITC).

☒ The axis scales are clearly visible. Include numbers along axes only for bottom left plot of group (a 'group' is an analysis of identical markers).

☒ All plots are contour plots with outliers or pseudocolor plots.

☒ A numerical value for number of cells or percentage (with statistics) is provided.

Methodology

Sample preparation	Ferret PBMCs were stimulated for 18-20 hours with 2 pools of SARS-CoV-2 spike peptides (S1-pool 1 and pool 2 or S2-pool 3 and pool 4) at a final concentration of 2µg/ml or ConA in the presence of golgi-stop (BD) and golgi-plug (BD). Cells were surface stained with anti-mouse/rat/human CD3 Alexa 405 (clone PC3/188A, 1 in 10 dilution) (Santa Cruz Biotechnology), anti-human CD8 APCCy7 (clone OKT8, 1 in 10 dilution) (Thermofisher) and live-dead aqua (Thermofisher), fixed with Fix-Perm solution prior to intracellular staining with anti-bovine IFNg PE (clone CC302, 1 in 10 dilution) (Abserotec) and anti-mouse TNFa A647 (clone MP6-XT22, 1 in 25 dilution).<br>Assays were performed using 50µl of heparinised blood incubated for 30 minutes at room temperature with optimal dilutions of the following antibodies: anti-CD3-AF700 (clone SP34-2, 1.25l per sample), anti-CD4-APC-H7 (clone L200, 10l per sample), anti-CD8-PerCP-Cy5.5 (clone SK1, 5l per sample), anti-HLA-DR-BUV395 (clone G45-5, dilution 2.5l), anti-CD25-FITC (clone M-A251, 20l per sample) (all from BD Biosciences, Oxford, UK); anti-CD14-PE (clone M5E2, dilution 5l) (Beckman Coulter); anti-CD16-BV785 (clone 3G8, dilution 5l), anti-CD20-PE-Dazzle (clone 2H7, 2.5l per sample), anti-CD95-PE-Cy7 (clone DX5, 5l per sample), anti-CD279(PD1)-BV711 (clone EH12-2H7, 5l per sample), anti-γδ-TCR-BV421 (clone TCR, 5l per sample) (all from BioLegend); and amine reactive fixable viability stain red (Life Technologies); all prepared in brilliant stain buffer (BD Biosciences). Red blood cell contamination was removed using a Utilyse reagent kit as per the manufacturer's instructions (Agilent). BD Compbeads (BD Biosciences) were labelled with the above fluorochromes for use as compensation controls. Following antibody labelling, cells and beads were fixed in a final concentration of 4% paraformaldehyde solution (Sigma Aldrich, Gillingham, UK) prior to flow cytometric acquisition. Cells were analysed using a five laser LSRII Fortessa instrument (BD Biosciences) and data were analysed using FlowJo (version 9.7.6, BD Biosciences).
Instrument	BD FortessaX2
Software	FACs DIVA software
Cell population abundance	*Describe the abundance of the relevant cell populations within post-sort fractions, providing details on the purity of the samples and how it was determined.*
Gating strategy	Ferret antigen specific T cells were identified by gating on LIVE/DEAD negative, doublet negative (FSC-H vs FSC-A), size (FSC-A vs SSC), CD3+, then CD4+ or CD8+ cells and IFNg+ (Figure S6).<br>NHP leukocyte populations were identified using a forward scatter-height (FSC-H) versus side scatter-area (SSC-A) dot plot to identify the lymphocyte, monocyte and granulocyte populations, to which appropriate gating strategies were applied to exclude doublet events and non-viable cells (Figure S7). Lymphocyte sub populations including T-cells, NK-cells, NKT-cells and B-cells were delineated by the expression pattern of CD3, CD20, CD95, CD4, CD8, CD127, CD25, CD16 and the activation and inhibitory markers HLA-DR and CD279 (PD-1). Classical- and non-classical-monocytes were identified by expression pattern of HLA-DR, CD14 and CD16. Granulocyte populations were delineated into neutrophils and eosinophils by expression of HLA-DR and CD14.

☒ Tick this box to confirm that a figure exemplifying the gating strategy is provided in the Supplementary Information.
